# Biosurfactants direct the evolutionary dynamics of surface-associated microbial systems

**DOI:** 10.1126/sciadv.aeh8127

**Published:** 2026-09-25

**Authors:** Chujin Ruan, Bijing Xiong, Deepthi P. Vinod, Anton Kan, Jing Wu, Madhav P. Thakur, David R. Johnson

**Affiliations:** ^1^ Department of Environmental Microbiology, Swiss Federal Institute of Aquatic Science and Technology (Eawag), Dübendorf, Switzerland.; ^2^ College of Ocean and Earth Sciences, Xiamen University, Xiamen, China.; ^3^ Department of Biosystems Science and Engineering, Swiss Federal Institute of Technology Zürich (ETH Zürich), Basel, Switzerland.; ^4^ Department of Environmental Systems Science, Swiss Federal Institute of Technology Zürich (ETH Zürich), Zürich, Switzerland.; ^5^ Department of Materials, Swiss Federal Institute of Technology Zürich (ETH Zürich), Zürich, Switzerland.; ^6^ School of Life Sciences, University of Science and Technology of China, Hefei, China.; ^7^ Institute of Ecology and Evolution, University of Bern, Bern, Switzerland.

## Abstract

Surface-associated microbial systems experience spatial constraints that can intensify stochastic lineage loss through genetic drift and alter how natural selection acts on competing lineages. A key feature of these systems is that steep resource gradients typically confine growth to a narrow band of cells located at the biomass periphery. This restricts the effective population size and constrains lineage interactions, thereby accelerating stochastic lineage exclusion. Here, we demonstrate that biosurfactants, which are secreted by many bacteria, can modulate the evolutionary dynamics of these systems by relaxing spatial constraints. Using *Stutzerimonas stutzeri* as a model bacterium, we used surface-associated growth experiments and individual-based simulations to demonstrate that rhamnolipid-induced reductions in surface friction widen the peripheral band of growing cells, consequently enhancing lineage intermixing. This relaxation of spatial constraints suppresses exclusion by genetic drift, mitigates competitive asymmetries, and increases the persistence of slower-growing variants. Our simulations reveal that reducing surface friction alone is sufficient to cause these effects, establishing a direct mechanistic link between spatial constraints and evolutionary outcomes in microbial systems. Our results demonstrate that biosurfactants not only promote ecological dispersal but also direct evolutionary dynamics by determining the spatial constraints acting on the system. Spatial constraints are therefore important determinants of microbial population dynamics and underappreciated regulators of evolutionary processes.

## INTRODUCTION

Microbial systems in natural, industrial, and clinical settings predominantly exist in surface-associated states, such as colonies, aggregates, and biofilms (*1*–*3*). The spatial organization of different microorganisms within these systems is a primary determinant of system dynamics and evolutionary trajectories (*4*–*6*), influencing competitive outcomes and the spread of genetic elements (*7*–*10*). A key feature of these surface-associated systems is that growth-supporting resources are typically replenished from the neighboring environment, such as the surrounding fluid, host tissue, or physical substratum, and enter the biomass along its periphery (*11*). Because these resources are rapidly consumed, only cells located within a narrow band positioned at the biomass periphery have access to them and can grow (referred to as the peripheral growth band) (*11*). This spatial constraint on resource supply substantially reduces the effective population size (*12*, *13*) (*N*_e_; defined as the number of individuals that successfully contribute genes to the next generation), thereby intensifying stochastic lineage loss through genetic drift and altering how selection acts on competing lineages. As a result, initially well-mixed populations rapidly segregate into relatively large clonal sectors (*14*, *15*), which limits short-range microbial interactions such as metabolite exchange (*16*, *17*), plasmid transfer (*18*–*20*), and contact-dependent antagonism (*21*, *22*). From an evolutionary perspective, low *N*_e_ at the biomass periphery strengthens genetic drift relative to selection, making it difficult for new variants to establish and slowing adaptive evolution (*23*–*25*). This reflects a core population genetic principle: Any factor that alters *N*_e_ can modulate the balance between random genetic drift and selection, thereby affecting both the speed and trajectory of evolution (*25*).

Beyond resource supply, mechanical interactions between cells and their environment shape microbial spatial organization at the biomass periphery (*26*, *27*). Within biofilms, interfacial mechanics (i.e., the physical forces and constraints acting at the cell-surface interface) govern how cells arrange themselves during growth and division (*28*). For example, extracellular matrix components mediate adhesion and frictional coupling to surfaces, thereby controlling the width of the peripheral growth band, patterns of cell orientation, and the formation of topological defects (*29*). Previous theoretical and experimental studies have established that mechanical interactions during surface-associated microbial growth can influence biomass morphology, the width of the actively growing layer, allele surfing, genetic drift, and the action of selection (*30*–*34*). In particular, surface friction and other mechanical constraints can regulate growth layer thickness and the surfing probability of beneficial mutations (*30*, *31*), while collective motion and cell crowding can modify apparent selection and the persistence of slower-growing clones or lineages (*32*, *33*). These studies provide an important physical and evolutionary framework for understanding how mechanical constraints shape surface-associated microbial systems.

Interfacial mechanics can be actively modified by microbial secretions that alter surface properties (*35*, *36*). Biosurfactants, which are amphiphilic molecules secreted by many bacteria, are widespread and important modulators of interfacial mechanics (*37*, *38*). Among these, glycolipids, such as rhamnolipids produced by *Pseudomonas* species (*39*–*42*) and other γ-proteobacteria, are commonly secreted and detected in soil (*43*), freshwater (*44*), marine (*45*), and plant-associated microbiomes (*46*). By lowering surface tension and reducing friction at cell-surface interfaces, biosurfactants not only promote biomass spreading and dispersal but also alter the spatial constraints acting at the biomass periphery (*42*). Despite their ubiquity and established ecological roles in soil aggregation (*47*), pollutant degradation (*48*, *49*), and biofilm dynamics (*50*–*52*), biosurfactants remain underrecognized as modulators of evolutionary processes, although biosurfactant-driven changes in interfacial mechanics could influence effective population size, genetic drift, and the strength of selection during surface-associated growth.

**Fig. 1.**
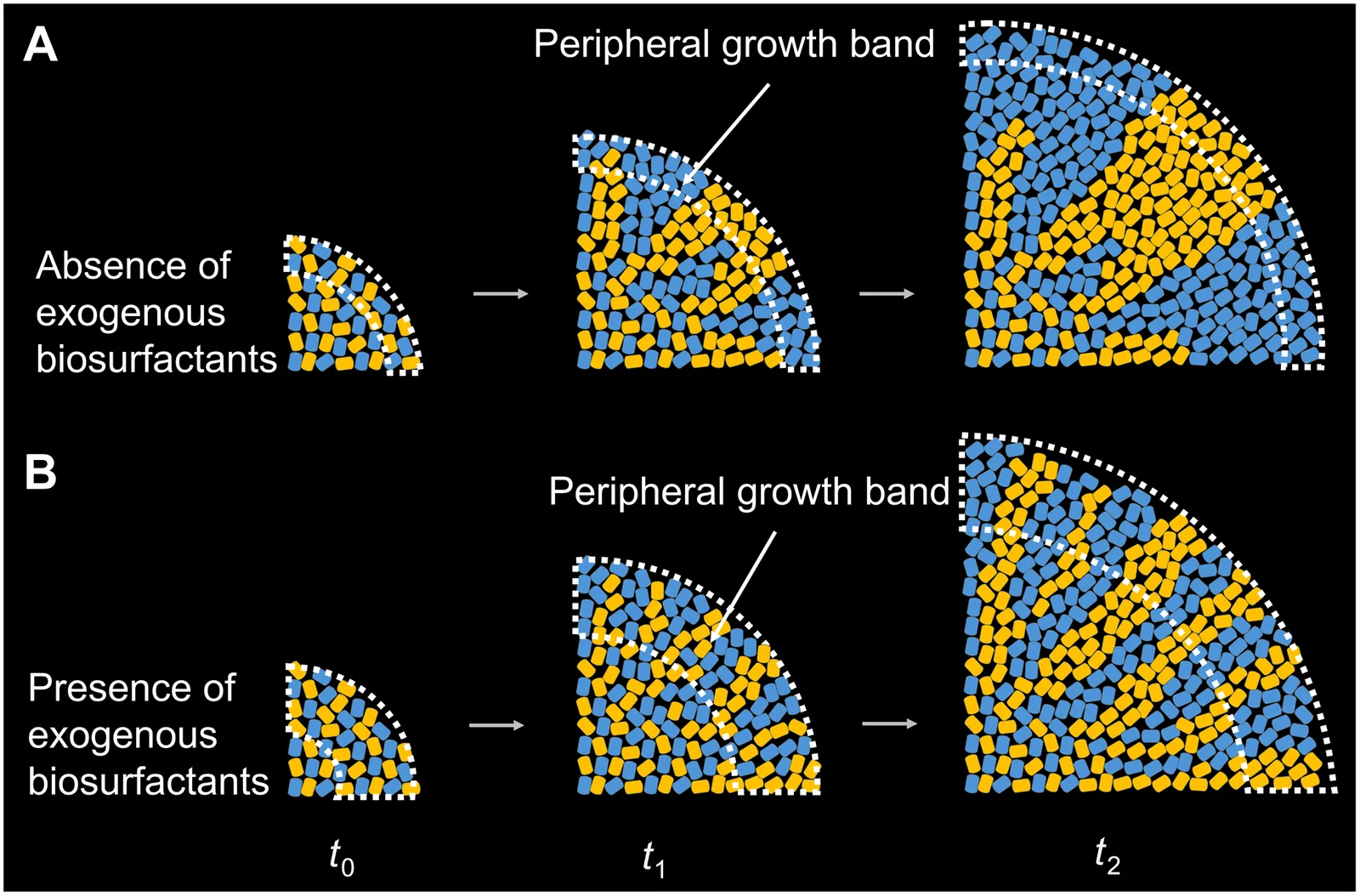
Schematic of the main hypothesis. (**A** and **B**) Conceptual illustration of how biosurfactants modulate ecological and evolutionary dynamics during surface-associated microbial growth. In the absence of biosurfactants, high surface friction restricts the outward displacement of interior cells, confining growth to a narrow peripheral growth band. This thin peripheral growth band reduces *N*_e_, intensifies random drift-driven lineage exclusion, and promotes the formation of relatively large sectors of conspecifics. Biosurfactants decrease frictional resistance by reducing surface friction, increasing the probability of interior cells reaching the biomass periphery and widening the peripheral growth band. This increases *N*_e_, enhances spatial intermixing (shown by interwoven lineage colors), and mitigates random drift-driven exclusion, thereby preserving biodiversity during surface-associated growth. Color-coded lineages identify distinct variants, and white dashed boxes delineate the peripheral growth band.

Here, we address this knowledge gap by testing the hypothesis that biosurfactant-mediated reductions in surface friction will relax spatial constraints and widen the peripheral growth band, consequently increasing *N*_e_ and shifting the evolution of surface-associated systems away from random genetic drift-dominated dynamics (Fig. 1, A and B). Because genetic drift can amplify the competitive exclusion of slower-growing lineages beyond what selection alone would predict, increasing *N*_e_ should weaken this effect and allow such variants to persist longer. We therefore reasoned that if biosurfactants weaken spatial constraints by reducing frictional coupling, then they should enhance lineage intermixing by slowing random genetic drift, mitigate the stochastic exclusion of lineages, and weaken competitive asymmetries that otherwise drive rapid clonal sorting (Fig. 1, A and B). We therefore predict that biosurfactants will affect multiple system properties: (i) physical, in the form of reduced frictional coupling and widening of the peripheral growth band; (ii) ecological, in the form of greater spatial intermixing at the biomass periphery; and (iii) evolutionary, in the form of increased persistence of slower-growing variants in the face of growth rate asymmetries.

To test this hypothesis, we performed experiments with isogenic fluorescently tagged strains of the bacterium *Stutzerimonas stutzeri* and quantified how exogenous biosurfactants affected lineage propagation and intermixing during surface-associated growth. We further introduced slower-growing tetracycline-resistant variants and quantified how exogenous biosurfactants affected their persistence in the face of faster-growing nonresistant counterparts. To achieve this, we combined confocal laser scanning microscopy (CLSM) with quantitative image analysis to resolve microbial spatial organization at the biomass periphery and single-cell time-lapse microscopy to visualize dynamics within the peripheral growth band. In addition, we used individual-based modeling to evaluate how reductions in surface friction alter the width of the peripheral growth band, competitive outcomes in the face of growth rate asymmetries, and the maintenance of lineage diversity. This comprehensive approach allows us to directly link interfacial spatial constraints to core population genetic parameters by connecting surface friction to *N*_e_, the intensity of random genetic drift, the efficacy of selection, and the speed and trajectory of evolution in surface-associated microbial systems.

## RESULTS

### Biosurfactants promote spatial intermixing

**Fig. 2.**
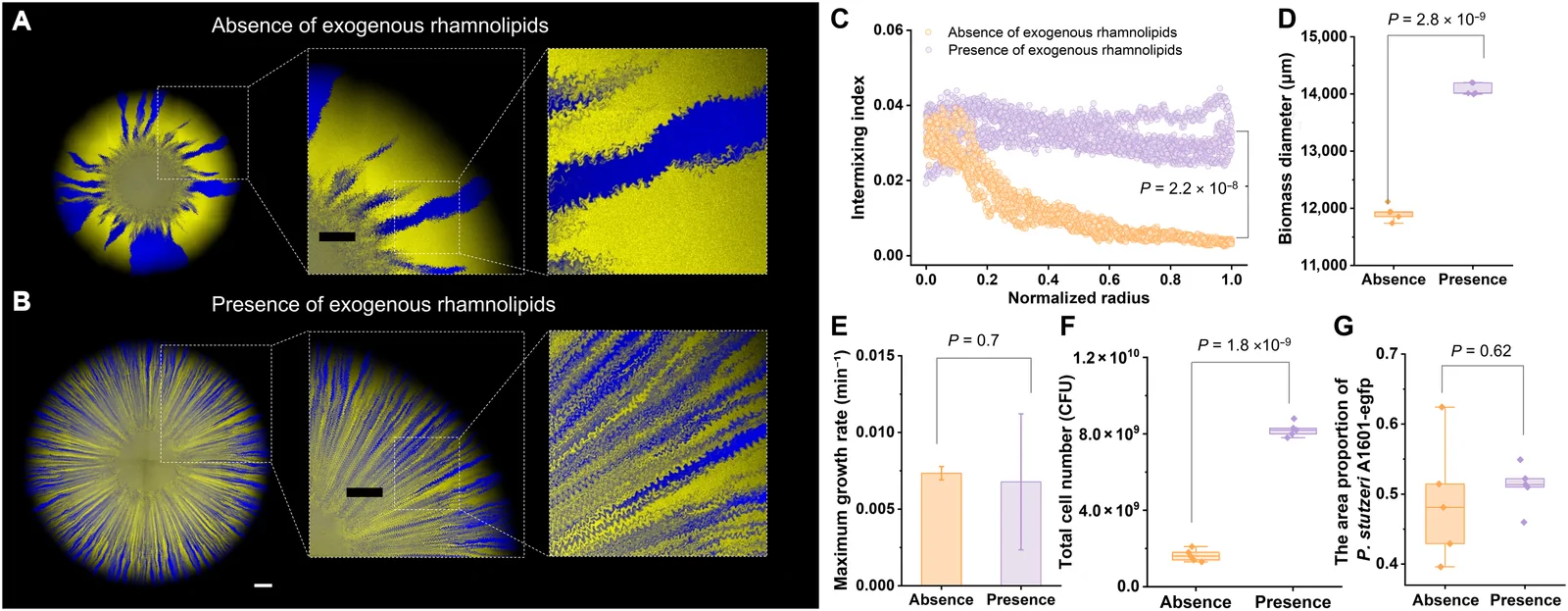
Rhamnolipids increase spatial intermixing during surface-associated microbial growth. (**A** and **B**) Representative CLSM images of isogenic fluorescently labeled strains of *S. stutzeri* (strains A1601-egfp and A1601-mcherry) coinoculated at a 1:1 ratio onto LB agar in the absence or presence of exogenous rhamnolipids, with GFP-expressing cells colored blue and mCherry-expressing cells colored yellow. Biomass was imaged after 4 days of incubation at 30°C. Insets are progressively magnified regions highlighting differences in spatial intermixing. Scale bars, 1000 μm. (**C**) Spatial intermixing for consortia grown in the absence or presence of exogenous rhamnolipids. Spatial intermixing was quantified as a function of normalized radial position. For statistical comparisons between conditions, intermixing values at the outermost normalized radius were used, yielding one independent intermixing metric per colony. (**D**) Final biomass diameters of consortia after 4 days of growth on agar surfaces. (**E**) Maximum growth rates of liquid consortia of strains A1601-egfp and A1601-mcherry in liquid LB medium with the same rhamnolipids treatments. (**F**) Quantification of total cell numbers [colony-forming units (CFU)] from consortia of *S. stutzeri* biomass grown on agar surfaces in the absence or presence of exogenous rhamnolipids. (**G**) Proportion of the biomass area occupied by strain A1601-egfp after growth in the absence or presence of exogenous rhamnolipids. For (C) to (G), each data point is an independent experimental replicate (*n* = 5), the orange data points are for experiments in the absence of exogenous rhamnolipids, and the purple data points are for experiments in the presence of exogenous rhamnolipids. For (D) to (G), the *P* values are for two-sample two-sided Welch tests. Source data are available in the repository described in the “Data, code, and materials availability” section.

To test the hypothesis that biosurfactant-induced reductions in surface friction alter the spatial organization of surface-associated microbial systems, we coinoculated isogenic fluorescently labeled variants of *S. stutzeri* (strains A1601-egfp and A1601-mcherry) at a 1:1 ratio onto lysogeny broth (LB) agar in the presence or absence of exogenous rhamnolipids and incubated them for 4 days at 30°C. The two strains are genetically identical except they express different fluorescent proteins, with green fluorescent protein (GFP) falsely colored blue and mCherry falsely colored yellow to aid visual differentiation. In the absence of exogenous rhamnolipids, we found that the initially well-mixed strains rapidly demixed into sectors of conspecifics, forming 20 ± 4 sectors radiating from the inoculation area (Fig. 2A and fig. S1). In the presence of exogenous rhamnolipids, in contrast, the strains generated substantially larger biomass diameters and a markedly finer pattern of sectors, indicative of enhanced strain intermixing (Fig. 2, B and C, and fig. S1). Quantitative analyses confirmed that the biomass diameters were significantly larger in the presence of exogenous rhamnolipids (two-sample two-sided Welch test; *P* = 2.8 × 10^−9^, *n* = 5) (Fig. 2D) as was spatial intermixing at the biomass periphery (two-sample two-sided Welch test; *P* = 2.2 × 10^−8^, *n* = 5) (Fig. 2C). These findings indicate that biosurfactants not only facilitate biomass spreading but also promote the intermixing of genetically distinct but functionally equivalent strains. To further assess the robustness and concentration dependence of this effect, we performed additional surface-associated growth experiments using different concentrations of rhamnolipids. Increasing the rhamnolipid concentration to 0.1% produced a spatial pattern consistent with that observed at 0.01%, with increased biomass spreading and spatial intermixing (fig. S2). In contrast, decreasing the concentration to 0.001% did not visibly increase spatial intermixing compared to experiments performed in the absence of rhamnolipids (fig. S2). In addition, Tween 20, a commonly used nonionic surfactant, produced a qualitatively similar effect to that observed with rhamnolipids, including increased biomass spreading and spatial intermixing (fig. S3). These observations further support the conclusion that surfactant-mediated changes in surface-associated growth can promote spatial intermixing.

To determine whether these differences in spatial intermixing caused by exogenous rhamnolipids arose from differences in intrinsic growth rates, we measured the maximum growth rates of consortia of strains A1601-egfp and A1601-mcherry in liquid LB medium in the presence or absence of exogenous rhamnolipids. We did not detect any significant differences in the maximum growth rates between the two conditions (two-sample two-sided Welch test; *P* = 0.7, *n* = 5) (Fig. 2E), indicating that rhamnolipids do not alter planktonic growth dynamics. However, in addition to increasing the biomass diameters (Fig. 2D), consortia grown on agar in the presence of exogenous rhamnolipids yielded substantially higher total cell numbers (measured as colony-forming units) than those grown in the absence of exogenous rhamnolipids (two-sample two-sided Welch test; *P* = 1.8 × 10^−9^; *n* = 5) (Fig. 2F). We attribute this increase not to increased cellular growth, but rather to rhamnolipid-mediated reductions in surface tension and friction that promote outward spreading and widening of peripheral growth band. This allows a larger fraction of cells to rapidly grow on the surface despite comparable intrinsic growth rates. When we quantified strain proportions as a function of biomass radius, we found that the relative abundances of the strains remained comparable between the two treatments (two-sample two-sided Welch test; *P* = 0.62, *n* = 5) (Fig. 2G), demonstrating that rhamnolipids do not alter strain dominance when the initial frequencies and growth rates are equal.

We next tested whether the effect of exogenous rhamnolipids on spatial intermixing is generalizable to other environments. We found that exogenous rhamnolipids also increased strain intermixing under anoxic conditions and at an alternative incubation temperature of 21°C (two-sample two-sided Welch test; *P* = 4.1 × 10^−4^, *n* = 5) (fig. S4), demonstrating that the qualitative effect is independent of oxygen availability and moderately lower temperatures. This effect was not restricted to *S. stutzeri*. We observed qualitatively similar outcomes when using isogenic fluorescently labeled strains of *Escherichia coli* (fig. S4), supporting the potential generality of this effect across phylogenetically distinct bacterial species.

### Rhamnolipids widen the peripheral growth band

**Fig. 3.**
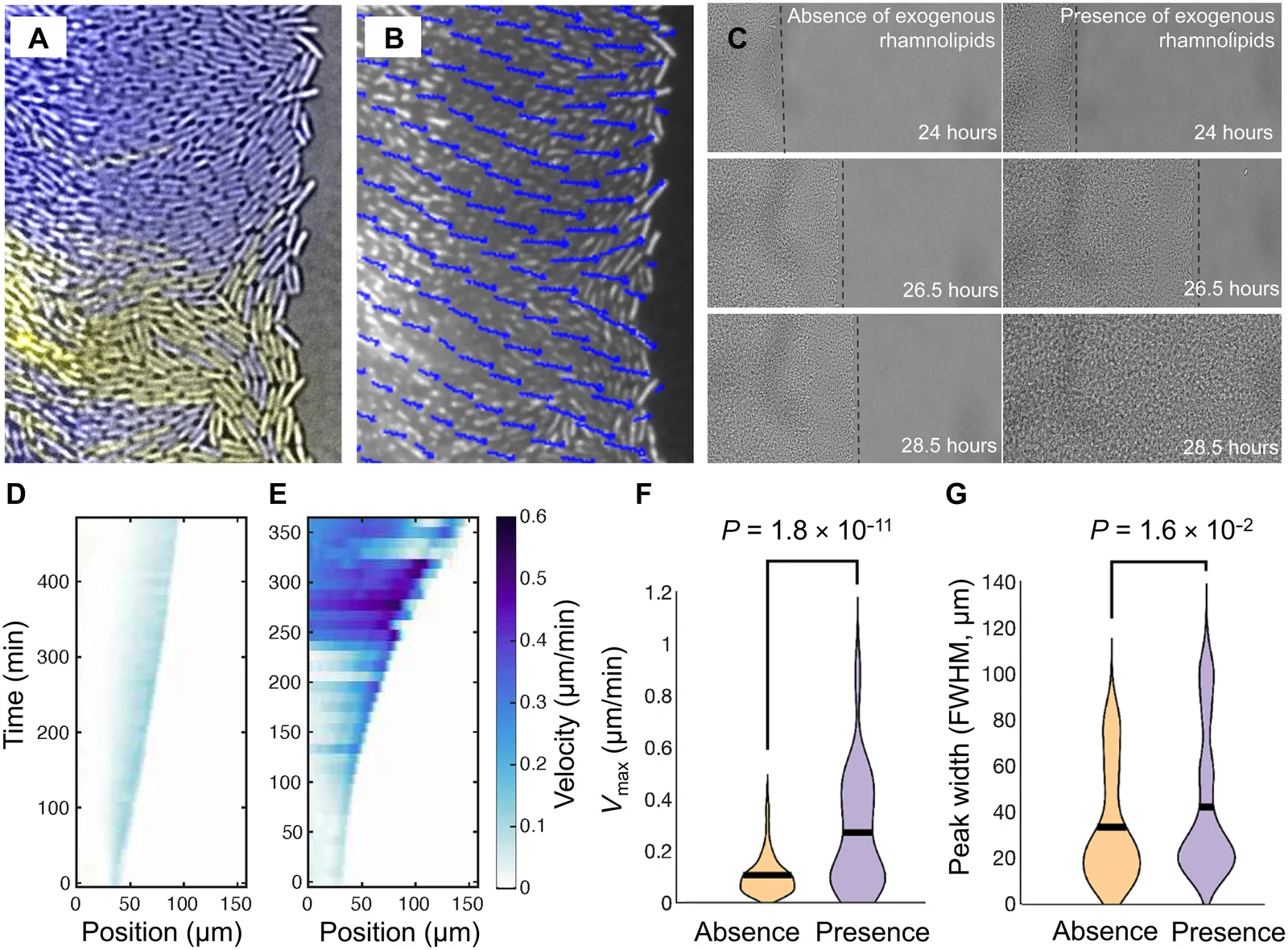
Exogenous rhamnolipids widen the peripheral growth band during surface-associated microbial growth. (**A**) Section of a time-lapse video of *S. stutzeri* cells showing the biomass periphery, with motion between consecutive frames quantified using a dense optical flow algorithm to generate the corresponding velocity field. (**B**) Postprocessed image of the same frame with the velocity field overlaid with blue arrows. (**C**) Peripheral growth band during growth on an agar surface either in the (left) absence or (right) presence of exogenous rhamnolipids. Time points indicate hours after inoculation. The dashed line represents the outer edge. (**D** and **E**) Kymographs of representative time-lapse videos during growth in the (D) absence or (E) presence of exogenous rhamnolipids, showing the velocity along the axis of biomass advancement. (**F** and **G**) The presence of exogenous rhamnolipids increased both (F) the maximum velocity and (G) the spatial width of the peak velocity distribution, indicating a widening of the peripheral growth band. Source data are available in the repository described in the “Data, code, and materials availability” section.

To directly quantify the impact of exogenous rhamnolipids on spatial organization and population dynamics during surface-associated growth, we performed single-cell time-lapse imaging of consortia of strains A1601-egfp and A1601-mcherry at a 1:1 initial ratio when the cells were embedded within agarose pads in the presence or absence of exogenous rhamnolipids. We visualized the advancing peripheral growth band over time and quantified its motion between consecutive frames using a dense optical flow algorithm (Fig. 3A). The resulting velocity field revealed coherent outward advancement of the peripheral growth band, corresponding to active cell growth, with velocity vectors overlaid in blue for clarity (Fig. 3B).

Kymographs of cell speed extracted along the axis of advancement of the peripheral growth band revealed a clear difference in the distributions of cell motion in the presence or absence of exogenous rhamnolipids (Fig. 3, C to E). In the absence of exogenous rhamnolipids, the biomass periphery displayed a relatively narrow band of high motion and reduced peak velocities (Fig. 3, C and D). In the presence of exogenous rhamnolipids, the biomass periphery displayed a markedly wider band of high motion, characterized by a more diffuse zone of high-speed cells (Fig. 3, C and E). Quantitative comparison confirmed that the presence of exogenous rhamnolipids increased both the maximum velocity and the extent of cell motion at the biomass periphery (Fig. 3, F and G). The maximal local velocity at the biomass periphery increased from ∼0.11 μm min^−1^ in the absence to ∼0.27 μm min^−1^ in the presence of exogenous rhamnolipids, with peak values approaching 0.93 μm min^−1^ (two-sample two-sided Welch test; *P* = 1.8 × 10^−11^, *n* = 3) (Fig. 3F). Concurrently, the full width at half maximum (FWHM) of the high-velocity band expanded from ∼33.7 μm in the absence to 42.5 μm in the presence of exogenous rhamnolipids, corresponding to an ∼26% widening of the peripheral growth band (two-sample two-sided Welch test; *P* = 1.6 × 10^−2^, *n* = 3) (Fig. 3G). These measurements demonstrate that exogenous rhamnolipids increase both the magnitude of local cell motion and the spatial extent of the high velocity region at the biomass periphery. Together, these data provide direct evidence that biosurfactants not only accelerate the absolute speed of biomass advancement but also widen the spatial domain of cell dispersal, thereby facilitating higher motion of cells during the early stages of biomass development.

### Surface friction determines the width of the peripheral growth band

**Fig. 4.**
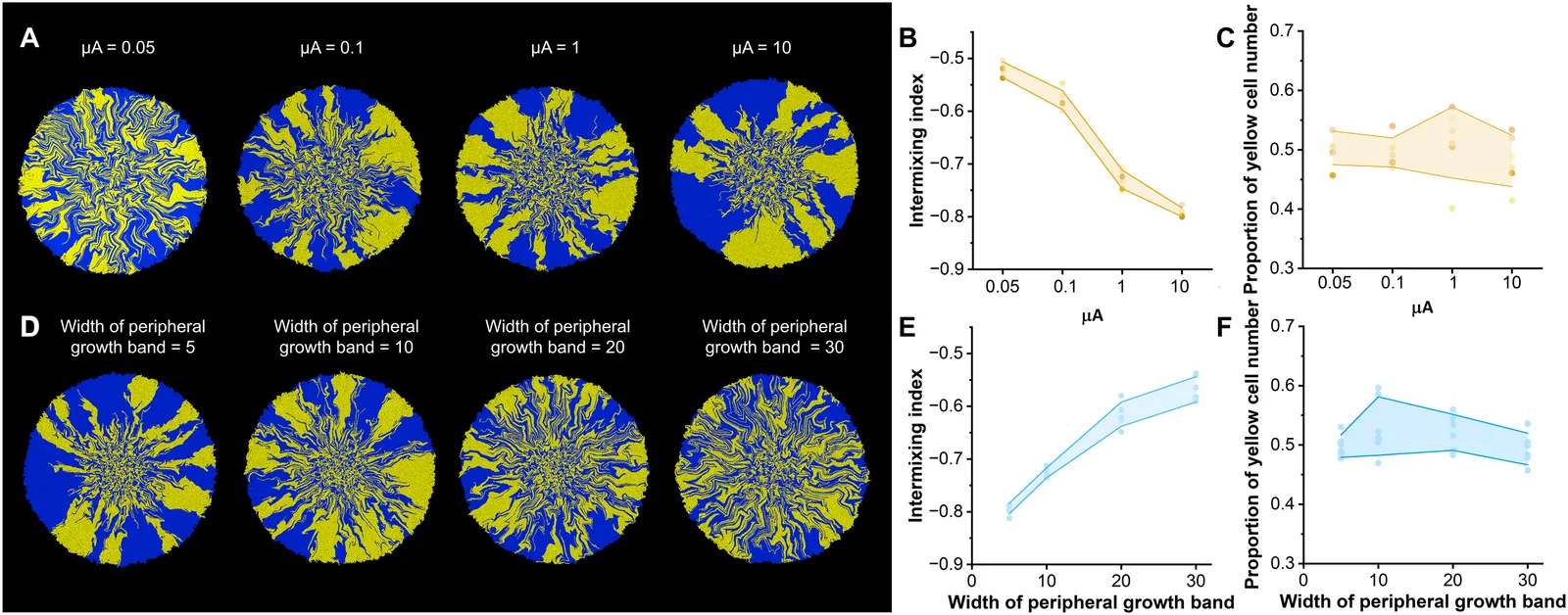
Surface friction and the width of the peripheral growth band determine spatial intermixing during surface-associated microbial growth. (**A**) Individual-based simulations of the effect of surface friction (μA) on spatial organization. Images are representative simulations of two cell types (yellow and blue) grown under different surface friction conditions (μA = 0.05, 0.1, 1, or 10). (**B** and **C**) Quantification of (B) the intermixing index and (C) the proportion of yellow cells from the simulations shown in (A) as a function of μA. (**D**) Individual-based simulations of the effect of the width of the peripheral growth band on spatial organization. Images are representative simulations with growth restricted to defined widths of the peripheral growth band (5, 10, 20, or 30 μm) under low surface friction conditions. (**E** and **F**) Quantification of (E) the intermixing index and (F) the proportion of yellow cells from the simulations shown in (D) as a function of width of peripheral growth band. For (B), (C), (E), and (F), each data point is an independent simulation (*n* = 6). Source data are available in the repository described in the “Data, code, and materials availability” section.

To dissect the mechanism underlying rhamnolipid-enhanced cell motion and spatial intermixing, we hypothesized that biosurfactants reduce surface friction, thereby relaxing spatial constraints and enhancing cell motion. To test this, we performed individual-based simulations in CellModeller (*53*, *54*), where our simulations explicitly consider cell mechanics and growth dynamics (Supplementary Text). For all our simulations, we deposited cells of two types at a 1:1 ratio to ensure that any differences in spatial patterning emerged solely from spatial effects rather than initial frequencies. By performing simulations across a range of surface friction values (μA = 0.05 to 10), we found that biomass growing under high surface friction conditions (μA = 1 or 10) developed relatively wide sectors of conspecifics (Fig. 4A), which is consistent with our experimental observations (Fig. 2A). In contrast, biomass growing under lower surface friction conditions (μA = 0.1 or 0.05) formed markedly narrower sectors of conspecifics with increased spatial intermixing (Fig. 4A), which is again consistent with our experimental observations (Fig. 2B).

Quantitative analyses revealed that the intermixing index significantly increased as surface friction decreased, with progressively higher intermixing observed at lower surface friction levels (Fig. 4B and fig. S5). There was also a strong negative association between surface friction and the intermixing index (Pearson correlation test: *r* = −0.98, *P* = 7.3 × 10^−16^, *n* = 6) (Fig. 4B). In contrast, the proportion of yellow cells was not statistically different across the surface friction conditions [analysis of variance (ANOVA) with a least significant difference Tukey post hoc test; *P* = 0.615, *n* = 6] (Fig. 4C and fig. S6), supporting the expectation that surface friction modulates spatial organization without affecting strain composition.

To investigate the mechanism underlying the dependence of spatial intermixing on surface friction, we hypothesized that reduced surface friction widens the peripheral growth band by allowing more interior cells to contribute to biomass propagation and advancement, which consequently increases *N*_e_. To test this, we performed constrained growth simulations where we restricted cell growth to a defined peripheral growth band while keeping surface friction constant (μA = 0.05). Increasing the width of the peripheral growth band from 5 to 30 μm led to a monotonic increase in spatial intermixing (Fig. 4D). There was also a strong positive relationship between the width of the peripheral growth band and the intermixing index (Pearson correlation test: *r* = 0.97, *P* = 1.7 × 10^−15^) (Fig. 4E and fig. S7). As with our previous simulations, the proportion of yellow cells remained largely unaffected across the tested widths of the peripheral growth band (ANOVA with a Tamhane post hoc test; *P* = 0.08, *n* = 6) (Fig. 4F and fig. S8), reinforcing the conclusion that spatial intermixing is primarily driven by a relaxation of spatial constraints rather than by changes in the cell type ratio. The experimental widening of the peripheral growth band (Fig. 3) closely mirrored the simulated widening of the peripheral growth band (Fig. 4), thereby unifying experimental and computational evidence. Collectively, these findings support a mechanism in which biosurfactants reduce surface friction, consequently widening the peripheral growth band and enhancing spatial intermixing at the biomass periphery.

### Exogenous rhamnolipids increase the persistence of slower-growing strains

To experimentally assess how surface friction influences competition between strains with different growth rates, we coinoculated strain A1601 with a tetracycline-resistant isogenic variant (A1601-Tc^R^) at a 1:1 initial ratio onto agar plates in the presence or absence of exogenous rhamnolipids as described for our previous experiments. After incubating the consortia for 4 days, we quantified and compared their spatial organization and strain persistence in the presence or absence of exogenous rhamnolipids.

**Fig. 5.**
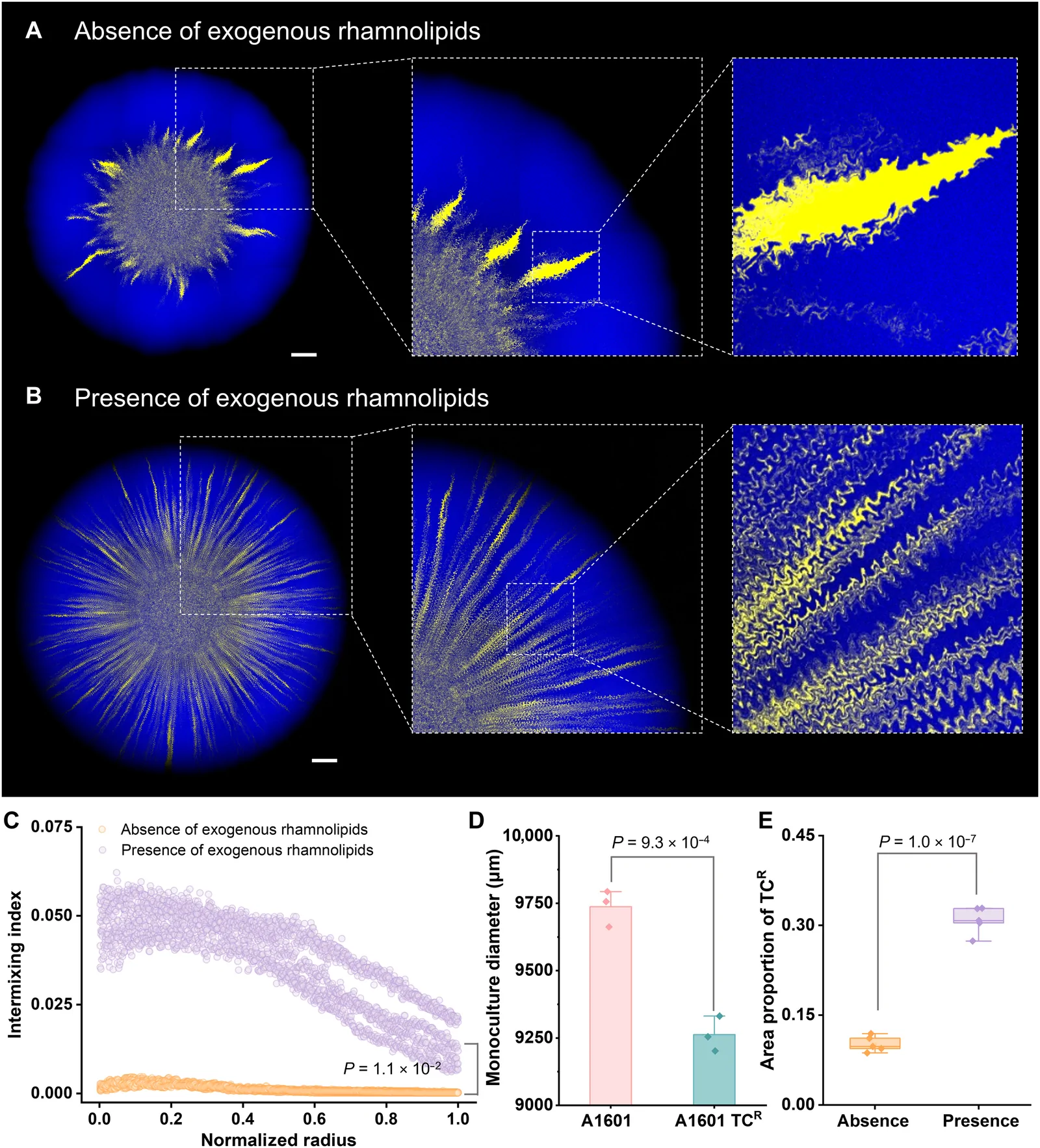
Rhamnolipids enable the persistence of a slower-growing strain. (**A** and **B**) Representative CLSM images of *S. stutzeri* consortia composed of strains A1601 (blue) and A1601-Tc^R^ (yellow), where the latter is a slower-growing isogenic tetracycline-resistant variant of the former. The consortia were inoculated at a 1:1 ratio on LB agar in the (A) absence or (B) presence of exogenous rhamnolipids. Images were acquired after 4 days of incubation. Insets are progressively magnified regions highlighting strain demixing in the absence of exogenous rhamnolipids and fine-scale intermixing in the presence of rhamnolipids. GFP-expressing and mCherry-expressing cells are colored blue and yellow, respectively. Images are representative of five independent biological replicates. Scale bars, 1000 μm. (**C**) Intermixing index as a function of the normalized biomass radius in the absence or presence of exogenous rhamnolipids. (**D**) Biomass diameters of strains A1601 and A1601-Tc^R^ when grown alone. (**E**) Proportions of the biomass areas occupied by strain A1601-Tc^R^ in the absence or presence of exogenous rhamnolipids in consortia. Source data are available in the repository described in the “Data, code, and materials availability” section.

Compared to consortia supplemented with rhamnolipids, consortia that were not amended with rhamnolipids appeared smaller in diameter and formed relatively large sectors of conspecifics, with strain A1601-Tc^R^ (yellow) forming sharply bounded sectors that did not persist at the biomass periphery (Fig. 5A). In the presence of exogenous rhamnolipids, by contrast, the consortia exhibited increased radial spreading and the formation of small narrow sectors of conspecifics that persisted at the biomass frontier (Fig. 5B). Quantification of spatial organization confirmed a significant increase in the intermixing index across the normalized biomass radius in the presence of exogenous rhamnolipids (two-sample two-sided Welch test; *P* = 1.1 × 10^−2^, *n* = 5) (Fig. 5C). Strain A1601-Tc^R^ grew more slowly than strain A1601 due to the tetracycline resistance phenotype, with strain A1601-Tc^R^ forming significantly smaller biomass diameters than strain A1601 (two-sample two-sided Welch test; *P* = 9.3 × 10^−4^, *n* = 5) (Fig. 5D). Consistently, the proportion of the total biomass area occupied by strain A1601-Tc^R^ increased from 10.18 ± 1.31% in the absence to 30.85 ± 2.24% in the presence of exogenous rhamnolipids (two-sample two-sided Welch test; *P* = 1.0 × 10^−7^, *n* = 5) (Fig. 5E). These results demonstrate that reductions in surface friction caused by exogenous rhamnolipids can promote spatial intermixing and enable the persistence of slower-growing strains despite their inherent growth disadvantage.

### Magnitude of the growth rate disadvantage determines the persistence of slower-growing cell types

**Fig. 6.**
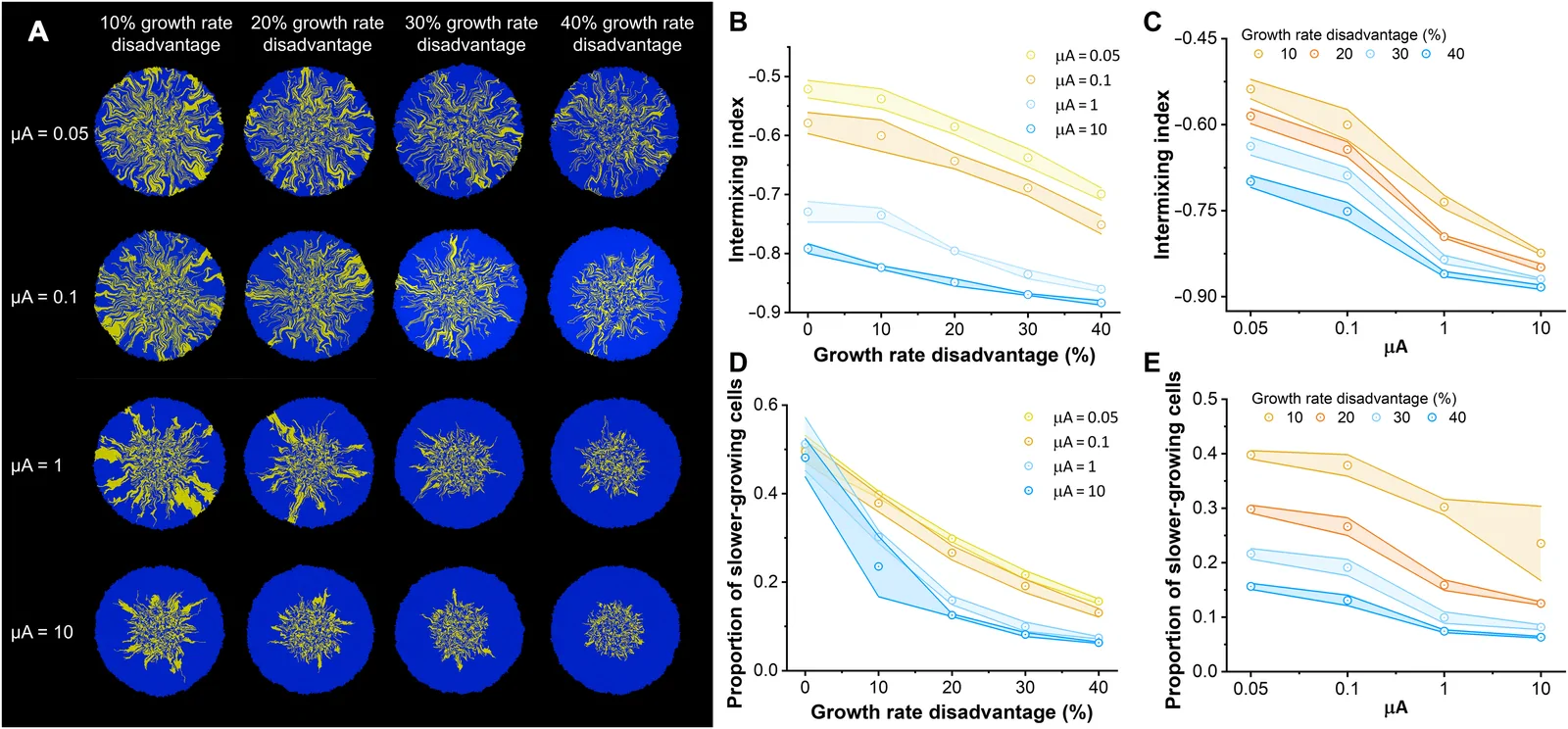
Surface friction and growth rate disadvantage jointly shape competitive outcomes during surface-associated growth. (**A**) Individual-based simulations of how the magnitude of the growth rate disadvantage of a slower-growing cell type affects its persistence for different surface friction (μA) levels. Simulations consisted of a reference (blue) and a slower-growing (yellow) cell type, where the growth rate of the latter was reduced by 10 to 40% relative to the reference. (**B** and **D**) Quantification of (B) the intermixing index and (D) the proportion of slower-growing cells as a function of their growth rate disadvantage for different surface friction levels. (**C** and **E**) Intermixing index (C) and proportion (E) of slower-growing cells as a function of surface friction for each magnitude of the growth rate disadvantage. For (B) to (E), each data point is an independent simulation (*n* = 6), lines connect the mean values, and shaded regions are ±1 SD. Source data are available in the repository described in the “Data, code, and materials availability” section.

To quantitatively evaluate how the magnitude of the growth rate disadvantage interacts with spatial constraints to shape spatial organization and competitive outcomes between different cell types, we performed individual-based simulations to quantify the effects of the intrinsic growth rate disadvantage for different levels of surface friction. Under high surface friction conditions (μA = 1 to 10), cells with lower growth rates were rapidly excluded from the biomass periphery, particularly when the growth rate disadvantage was high. Under low surface friction conditions mimicking biosurfactant supplementation (μA = 0.05 to 0.1), in contrast, cells with mild growth rate disadvantages could form sectors of conspecifics that persisted at the biomass periphery (Fig. 6A). In general, spatial intermixing increased as surface friction decreased, indicating that the relaxation of spatial constraints systematically increases the persistence of slower-growing cell types by attenuating selective exclusion (Fig. 6A). These qualitative observations are supported by quantitative measurements. The intermixing index declined significantly as the growth rate disadvantage of the slower-growing cell type increased regardless of surface friction conditions (Spearman rank correlation test; ρ = −0.94, *P*_BC_ = 3.2 × 10^−14^, *n* = 6) (Fig. 6B and fig. S9). For each magnitude of the growth rate disadvantage, lower surface friction resulted in markedly higher spatial intermixing. In general, we observed a negative relationship between surface friction and the intermixing index as the growth rate disadvantage of the slower-growing cell type increased from 10 to 40% (Spearman rank correlation test; ρ = −0.97, *P*_BC_ = 3.7 × 10^−14^, *n* = 6) (Fig. 6C).

We observed a similar trend for the composition of cell types. The proportion of slower-growing cells (yellow) decreased sharply as its growth rate disadvantage increased (Spearman rank correlation test; ρ = −0.98, *P*_BC_ = 1.1 × 10^−20^, *n* = 6) (Fig. 6D and fig. S10), with steeper decreases for higher surface friction. Surface friction also negatively correlated with the proportion of slower-growing cells (Spearman rank correlation test; ρ = −0.91, *P*_BC_ = 3.6 × 10^−9^, *n* = 6) (Fig. 6E). Together, these results demonstrate that while reduced surface friction promotes spatial intermixing and delays competitive exclusion of slower-growing cell types, the magnitude of the growth rate disadvantage ultimately governs the long-term persistence of slower-growing cell types at the biomass periphery.

### Lower surface friction slows the loss of cell type diversity

We finally tested whether the effect of surface friction on the maintenance of slower-growing cell types extends to surface-associated systems containing larger numbers of cell types with different growth rates. To test this, we extended our individual-based model to simulate surface-associated systems composed of three to six cell types that differed only in their intrinsic growth rate. Each consortium was simulated under either low or high surface friction conditions, yielding a total of 297 matched simulation pairs that enabled direct pairwise comparisons of consortia outcomes. At the end of each simulation, we quantified cell type diversity by calculating diversity metrics that consider the relative abundances of growth rate–defined cell types, including Shannon entropy and Pielou’s evenness.

**Fig. 7.**
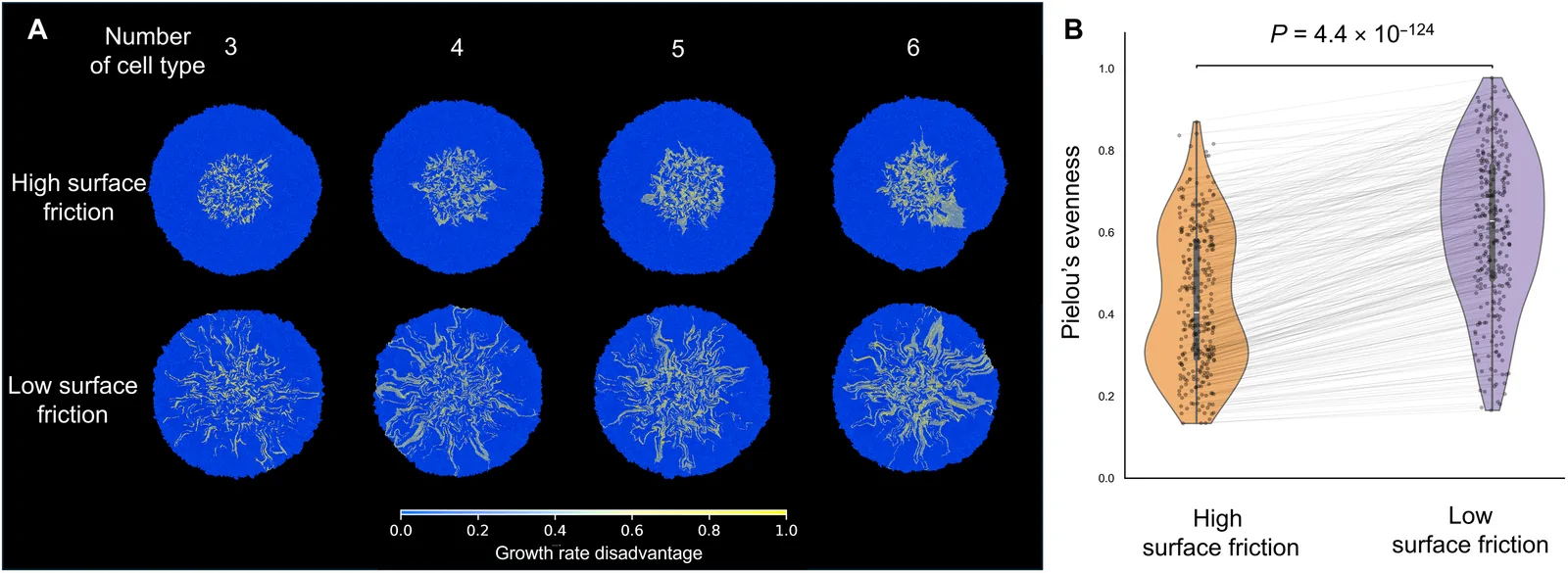
Surface friction determines the maintenance of cell type diversity. (**A**) Representative individual-based simulations of consortia consisting of three to six distinct cell types growing under (top) high surface friction (μA = 10) or (bottom) low surface friction (μA = 0.05) conditions. Each cell type was assigned a growth rate from 0 to 1 by randomly sampling from a uniform distribution. Cell colors indicate cell types with different growth rates ranging from blue (growth rate = 0) to yellow (growth rate = 1). (**B**) Pielou’s evenness of simulated consortia growing under (left) high surface friction or (right) low surface friction conditions. Violin plots show the distribution of evenness values, with the overlaid boxplots indicating the median and interquartile range. Each data point is for an individual simulation, and gray lines connect paired simulations for low and high surface friction (*n* = 297 pairs). The *P* value is for a two-sided paired *t* test. Source data are available in the repository described in the “Data, code, and materials availability” section.

Regardless of the initial number of cell types, reducing surface friction consistently preserved cell type diversity in the face of growth rate differences. Consortia simulated under low surface friction conditions exhibited more even relative abundances of the different cell types than those simulated under high surface friction conditions (Fig. 7A). The mean Pielou’s evenness of cell type abundances was consistently higher under low surface friction conditions across matched simulation pairs (paired two-sided *t* test, *P* = 4.4 × 10^−124^, *n* = 297 pairs) (Fig. 7B). We also confirmed that the increase in cell type diversity under low surface friction conditions was consistent regardless of the initial number of cell types. Shannon entropy (paired two-sided *t* test, *P*_BC_ = 9.1 × 10^−24^, *n* = 67 to 87 pairs per richness level) and Pielou’s evenness (paired two-sided *t* test, *P*_BC_ = 9.1 × 10^−24^, *n* = 67 to 87 pairs per richness level) were always significantly greater under low surface friction conditions compared to high surface friction conditions (fig. S11). Together, these findings demonstrate that a relaxation of spatial constraints via lower surface friction enables cell types with different growth rates to persist, thus maintaining cell type diversity during surface-associated microbial growth.

## DISCUSSION

Our results demonstrate that biosurfactants can direct evolutionary dynamics by modulating a central population genetic parameter, the effective population size (*N*_e_) (*12*, *13*). By increasing *N*_e_, biosurfactants slow genetic drift, mitigate the stochastic exclusion of lineages, and extend the period over which weak selection can act on fitness differences. In surface-associated microbial systems, growth and division are confined to a narrow peripheral band where drift is intensified and diversity is rapidly lost (*23*, *24*, *55*). Surface friction emerges here as a fundamental spatial determinant of evolutionary outcomes. The biosurfactant-induced reduction in surface friction widens the peripheral growth band, allowing interior cells to participate in outward growth and thereby increasing the effective number of reproducing individuals. This relaxation of spatial constraints transforms the nature of competition at the biomass periphery from a process dominated by genetic drift to one where weak selective differences can persist. Experimental evidence of enhanced spatial intermixing, supported by simulations across a continuum of surface friction values, provides a mechanistic link between the biophysics of cell-surface interactions and the population genetics during surface-associated growth. This effect is likely to depend on sufficient biosurfactant concentrations: Only when surfactant accumulation adequately reduces surface friction, it can enhance spatial intermixing and downstream evolutionary shifts become detectable. In this view, biosurfactants can modify the physical context of growth, thereby altering the spatial constraints that shape selective and evolutionary dynamics. Our findings are consistent with previous work showing that environmental heterogeneity, substrate geometry, mechanical interactions between cells and the substrate, surface friction, and the thickness of the actively growing layer can influence spatial sorting, allele surfing, genetic drift, and the action of selection during surface-associated microbial growth (*30*–*34*, *56*, *57*). The actively growing layer described in these studies is conceptually related to the peripheral growth band examined here (*30*–*33*). We extend these well-established physical and evolutionary frameworks to biosurfactants, a widespread class of microbial secretions that can modify interfacial mechanics (*37*, *40*). This extends the recognized physiological and ecological functions of biosurfactants to population genetic processes during surface-associated growth. By using exogenous rhamnolipids, we isolated the effect of reduced surface friction and showed that this physical change widens the peripheral growth band, enhances spatial intermixing, and delays the exclusion of slower-growing variants in surface-associated microbial systems. Thus, our results identify rhamnolipids as potential modulators of evolutionary processes during surface-associated growth.

The recognition that biosurfactants modulate *N*_e_ through the relaxation of spatial constraints reframes their previously recognized ecological functions. Traditionally viewed as promoters of motility (*58*), dispersal (*37*), or virulence (*51*), our findings confirm that biosurfactants also act as mediators of evolutionary opportunity, subtly altering how genetic drift and selection unfold in surface-associated systems. This multifunctional role may help motivate future tests of whether evolutionary benefits linked to altered spatial constraints contribute to the maintenance of costly biosurfactant production in some taxa; by increasing *N*_e_ and prolonging persistence (*6*), biosurfactant-mediated changes in surface mechanics could preserve diversity upon which selection can act. In ecological contexts, this mechanism may enhance the stability of polymorphic populations or multispecies consortia by preventing early stochastic exclusion (*59*). Conversely, in pathogenic or engineered systems, increased spatial intermixing can accelerate the horizontal spread of resistance or virulence genes (*7*, *16*).

The relaxation of spatial constraints by lowering surface friction not only changes the importance of genetic drift but also modifies how selection acts on competing strains. When surface friction is high, spatial confinement at the biomass periphery can accelerate the loss of slower-growing variants by combining deterministic growth rate differences with stochastic lineage loss. When surface friction is low, the wider peripheral growth band allows more lineages to propagate, which reduces rapid exclusion and prolongs the persistence of slower-growing variants. As shown in Fig. 5, the observed persistence of a tetracycline-resistant but slower-growing strain in the presence of exogenous rhamnolipids exemplifies this principle; relaxation of spatial constraints sufficiently buffers the strength of selection to sustain prolonged persistence. These results provide a possible mechanism by which costly antibiotic resistance traits may persist transiently during surface-associated growth in the absence of antibiotic pressure (*60*). However, this buffering has limits, as sufficiently large growth rate differences eventually overcome the benefits of relaxed spatial constraints (Fig. 6), indicating that the relaxation of these constraints can delay but not abolish competitive exclusion. These findings help extend theoretical predictions from evolutionary models of surface-associated microbial growth to real microbial systems, demonstrating that mechanical properties of cells and the surfaces over which they grow directly tune the balance between genetic drift and selection.

Beyond competition between pairs of strains, the relaxation of spatial constraints by lowering surface friction identifies a plausible biophysical mechanism that can contribute to the maintenance of diversity in some surface-associated microbial systems (*61*, *62*). Individual-based simulations incorporating multiple cell types revealed that lowering surface friction consistently increases both the Shannon entropy and evenness of surface-associated systems regardless of their initial composition (Fig. 7 and fig. S11). The widening of the peripheral growth band increases the number of copropagating lineages and reduces the dominance of faster-growing cell types, increasing persistence across a wide range of parameter combinations. These results suggest that biosurfactants may, under some conditions, promote the persistence of diverse lineages by relaxing spatial constraints; by reducing surface friction, they maintain the persistence of diversity and promote the intermixing necessary for short-range metabolic exchange (*16*, *17*, *63*), antagonism (*21*, *64*), and plasmid transfer (*18*–*20*, *65*), which are processes that depend on close spatial proximity among distinct lineages. In natural systems such as soil particles (*47*), biofilms (*42*), or marine aggregates (*45*), the secretion of biosurfactants could, in principle, contribute to maintaining spatial connectivity, where biosurfactant production or accumulation modifies cell-surface mechanics in the face of spatial constraints.

Although our combined experimental and computational approach reveals a robust link among surface friction, *N*_e_, and evolutionary processes, several limitations should be acknowledged. First, our study relies primarily on well-controlled laboratory conditions with a minimal set of microbial strains. While this approach reduces confounding factors and improves our ability to draw robust conclusions, it does not reflect the enormous biological complexity and environmental heterogeneity that can be present in many natural surface-associated microbial systems. It is important to test whether our observed principles continue to hold as system complexity and heterogeneity increase. Second, we used exogenous rhamnolipids rather than relying on endogenously produced rhamnolipids. We therefore demonstrate the mechanistic consequences of reduced surface friction, but not the full eco-evolutionary feedback whereby rhamnolipid-producing cells can reshape their own selective environment. Future experiments comparing biosurfactant-producing and nonproducing strains in more complex microbial communities will be needed to test whether endogenous biosurfactant production is sufficient to promote spatial intermixing under ecologically relevant conditions. Third, while our individual-based simulations incorporate spatial constraints with high spatial resolution, they do not account for dynamic feedbacks between biosurfactant production, nutrient availability, or the evolving mechanical properties of the surface that the cells associate with. Last, our work spans short to medium timescales, and the long-term evolutionary and ecological consequences of surface friction in strain persistence and diversity remain to be tested in systems where additional processes such as mutation, cooperation, or antagonism are present.

In conclusion, our findings highlight an evolutionary role of biosurfactants in modulating spatial constraints during surface-associated growth and highlight the importance of incorporating biophysical processes into evolutionary ecology. Spatial constraints such as those emerging from surface friction act as a continuous and tunable parameter that determines the strength of genetic drift, selection, and diversity across space and time. By reducing surface friction, biosurfactants increase *N*_e_, shift the dominance of evolutionary processes, and reshape eco-evolutionary outcomes. Embedding quantitative measures of surface friction into spatially explicit evolutionary models may reveal general principles by which organisms modify their local environment (e.g., niche construction) to control the evolutionary tempo of their populations. Such a framework generates testable hypotheses about how spatial constraints may influence the persistence of costly traits, such as antibiotic resistance, and the maintenance of diversity during surface-associated growth in several types of microorganisms.

## MATERIALS AND METHODS

### Bacterial strains

Isogenic variants of *S. stutzeri* A1601 (*66*, *67*), designated strains A1601-egfp and A1601-mcherry, were used to assess the effects of biosurfactants on microbial spatial organization and diversity during surface-associated growth. These strains are genetically identical except that they carry different chromosomally integrated fluorescent protein–encoding gene cassettes [either the green (GFP) or red (mCherry) fluorescent protein–encoding gene under the control of an IPTG-inducible *Ptac* promoter], enabling the strains to be distinguished and tracked over space and time when grown in consortia (*16*, *20*). The expression cassettes were inserted at the chromosomal Tn7 attachment site via a mini-Tn7T-LAC-Gm transposon delivered by a conditionally replicative pUC18T plasmid (*68*).

Tetracycline-resistant variants of strain A1601-mcherry were obtained by serially passaging stationary-phase cultures on LB agar supplemented with tetracycline in stepwise increments of 2 μg ml^−1^, beginning at 2 μg ml^−1^ and ending at 16 μg ml^−1^, over eight successive passages. At each step, single colonies from the highest concentration supporting visible colony formation after 24 hours at 30°C were streaked onto LB agar containing the same tetracycline concentration before proceeding to the next step. All strains were stored in 15% (v/v) glycerol at −80°C. Before each experiment, each strain was streaked from its frozen stock onto LB agar containing the relevant antibiotic, and single colonies were used to initiate overnight cultures. To quantify the fitness cost of tetracycline resistance, each strain was grown individually in LB broth in the absence of tetracycline, and optical density at 600 nm (OD_600_) was recorded at 15-min intervals for 24 hours using a BioTek/Agilent Synergy H1 microplate reader.

### Surface-associated growth assays

Surface-associated growth assays were performed on LB agar (1.5% w/v) in the presence or absence of exogenous rhamnolipids (lot no. A797462100) (AGAE Technologies, Corvallis, OR, USA). A 10% (w/v) stock solution of rhamnolipids was first filter-sterilized and incorporated into molten LB agar cooled to ∼60°C after autoclaving to achieve a final concentration of 0.01% (w/v). The medium was then dispensed into 60-mm-diameter petri dishes, allowed to solidify overnight at room temperature, air-dried for 10 min in a sterile laminar flow hood, sealed with Parafilm, and stored at 4°C until use.

For inoculum preparation, single colonies of strains A1601-egfp and A1601-mcherry were picked from freshly streaked LB agar and grown separately overnight in LB broth at 37°C with shaking at 200 rpm. Cultures were adjusted to an OD_600_ of 1.0 and mixed at a 1:1 ratio before inoculation (*18*, *64*). Because rhamnolipids alter the surface tension of agar, inoculation procedures were adjusted to ensure equal initial colony size and cell numbers across treatments. In the absence of exogenous rhamnolipids, 1 μl of the 1:1 mixture was spotted at the center of an LB agar plate. In the presence of exogenous rhamnolipids, 1 ml of the same mixture was centrifuged and resuspended in 0.2 ml of phosphate-buffered saline to achieve a fivefold concentration, and 0.2 μl of this suspension was spotted at the center of an LB agar plate. This volumetric adjustment yielded an initial droplet diameter of ∼2.7 mm and ensured equivalent initial cell numbers across the treatment conditions. The LB agar–containing plates were incubated under oxic conditions with the incubation temperature and duration optimized for each specific experiment as detailed in Results. Each experiment was performed with five independent biological replicates.

### Single-cell imaging of growing surface–associated biomass

Single-cell imaging of growing surface–associated biomass was performed on a circular agarose pad (18 mm in diameter) following procedures described by Young *et al.* (*69*). Ten ml of lukewarm LB medium containing 1.5% low-melting point agarose (Carl Roth, Karlsruhe, Germany) was first prepared. Next, 10 μl of 10% (w/v) rhamnolipids was added to the 1.5% agarose medium, and 0.25 ml of the lukewarm agarose medium was placed on a circular glass support (18 mm in diameter, ibidi, Gräfelfing, Germany). Immediately thereafter, another circular glass cover slide (18 mm in diameter) was placed on top of the deposited agarose, and the “sandwiched” agarose was allowed to cool for 10 min. The agarose pad was then flipped over, and the circular glass support was removed using tweezers. To inoculate the agarose pad, 1 μl of the bacterial mixture (OD_600_ = 0.1) was pipetted onto the surface of the agarose pad. The agarose pad was then allowed to dry for 15 min and flipped over and attached to a glass-bottom petri dish (35-mm μ-Dish; ibidi, Gräfelfing, Germany) so that the cells were sandwiched between the glass bottom of the petri dish and the glass cover–shielded agarose pad. To prevent evaporation and possible position shifts of the agarose pad during time-lapse microscopy, the entire petri dish was filled with lukewarm LB agar medium (1.5% agarose amended with 0.01% rhamnolipids), and the medium was allowed to cool and solidify for 1 hour. The prepared petri dish was then incubated for 12 hours in an oxic chamber at 30°C. Microscopic images of the cells were taken with an inverted microscope (Eclipse 2, Nikon, Japan) with a 100× oil immersion objective. Bright-field and fluorescence images were taken at 10-min intervals for 6 hours to continuously track GFP- and mCherry-expressing cells. Three replicates were performed in each condition.

### Optical flow analysis

Time-lapse images of growing biomass were analyzed using a custom Python (2.7.18) script that uses a dense Optical Flow algorithm to capture the motion of single cells (*70*). For preprocessing, the three-channel 16-bit time-lapse videos were manually combined into single 8-bit videos and down-scaled to a resolution of 600 × 600 pixels. The videos were analyzed using the Farneback Optical Flow algorithm (*71*) (cv.calcOpticalFlowFarneback) in the OpenCV (2.4.12) Python package, which generated a time-resolved vector field representing the motion of each region of the image between adjacent frames of the video. For each time-lapse video, the velocity field output was saved as an “.nc” file for subsequent analysis. To analyze the velocity field, a further script extracted the direction of bulk motion (or growth) by identifying the average vector for all velocities within the time-lapse video and rotating the image to align the direction of bulk motion with the *x* axis. Then, the velocity along the axis of bulk motion was averaged along the *x* axis for each frame, and the maximum velocity (*V*_max_) and the width of the velocity peak were determined by finding the peak FWHM.

The scripts were written in Python (v2.7.18) with the following dependencies: OpenCV (v2.4.12), NumPy (v1.9.3), scikit-image (v0.13.1), tifffile (v0.15.1), xarray (v0.10.0), SciPy (v1.1.0), and Matplotlib (v2.2.3). The cv2.calcOpticalFlowFarneback function was executed with the following parameters: pyr_scale = 0.5, levels = 4, winsize = 27, iterations = 20, poly_n = 5, poly_sigma = 1.5, and flags = cv2.OPTFLOW_FARNEBACK_GAUSSIAN.

### CLSM and image analysis

Surface-associated microbial biomass was imaged using a Leica Stellaris 5 confocal laser scanning microscope (Leica Microsystems, Wetzlar, Germany) built on a Leica DMi8 inverted stand. Images were acquired with a 2.5× HC PL FLUOTAR objective (numerical aperture, 0.07), yielding a final resolution of 1024 × 1024 pixels. Fluorescence excitation was provided by an integrated white light laser (485 to 685 nm, 85% power) and a 448-nm diode laser. GFP signals were excited at 488 nm and mCherry signals at 587 nm, with emission collected on HyD detectors set to 500 to 550 nm for GFP and 600 to 750 nm for mCherry. Image acquisition and processing were carried out using the Leica LAS X software platform. For further analysis, images were quantified using ImageJ (https://imagej.nih.gov/ij/) with Fiji plugins (v. 2.1.0/1.53c; https://fiji.sc). Image files were imported, and fluorescence channels were separated for the analysis of individual strains. At defined radial distances from the inoculation droplet periphery, a circle was drawn, and the number of color transitions (each strain expressing a different fluorescent protein) along the circumference was determined and normalized by the circumference. This analysis was repeated at multiple radii to obtain spatial intermixing as a function of radial distance from the biomass centroid. Before analysis, images were thresholded and binarized using the “Mean” algorithm, and noise was removed using the “Remove Outliers” function (radius = 2, threshold = 50, bright). Concentric windowing from the inoculation droplet edge to the biomass periphery was performed at 5-μm radial increments using the Sholl plugin (*72*). The intermixing index at each radius (*I*_r_) was calculated as the number of observed color transitions (*N*_r_) divided by the expected number of color transitions for a random spatial distribution of two strains at that radius (*5*, *16*, *73*), as defined in Eq. 1

Ir=Nrπr2 (1)

### Individual-based computational modeling

Individual-based simulations of surface-associated microbial growth were performed using the open-source modeling framework CellModeller (*21*, *22*, *53*, *54*) (version 4.3; https://github.com/cellmodeller/CellModeller). Simulations were run on the MicrobialEcologyToolbox branch and modified to investigate the effects of biosurfactant-mediated changes in surface friction on microbial spatial organization, competition between cell types, diversity of cell types, and the structure of the peripheral growth band during surface-associated growth.

Simulations were conducted in a quasi–two-dimensional annular domain (radius = 50 μm) to approximate biomass growth across a flat surface. Initial cell positions were randomly assigned using a custom placement function (randomXY), which seeded cells either within the inner circular core or in the annular region, while a nonoverlap constraint (randomNonOverlap) prevented unrealistic crowding of cells. Cells were modeled as rod-shaped agents with an initial length of 2.0 μm and a radius of 0.4 μm. Division thresholds were sampled from a Gaussian distribution. Exponential growth was implemented with binary fission triggered when a cell’s volume exceeded its division threshold, with daughter cells inheriting parental properties upon division. Mechanical interactions between individual cells and between cells and the surface were resolved using the spring-based force model of the CLBacterium biophysical module (*53*, *54*). Surface friction was parameterized by adjusting the surface friction (μA) between 0.05 and 10, with lower μA values simulating reduced friction and promoting local cell sliding. This parameterization provided a mechanistic representation of how changes in surface friction influence interfacial mechanics during surface-associated growth. The description of the surface friction in the model is provided in the Supplementary Materials.

Four simulation scenarios were designed, each targeting a specific aspect of surface-associated growth dynamics under varying surface friction, to systematically test mechanistic hypotheses regarding interactions between spatial constraints, growth rate asymmetries, diversity maintenance, and the spatial organization of the peripheral growth band. (i) In the first scenario, competition between two cell types with identical intrinsic growth rates was quantified as a function of μA to assess how surface friction modulates spatial organization. Simulations were initiated with equal numbers of cells of two cell types, and the emergent spatial patterns were compared across μA values. (ii) In the second scenario, the interaction between surface friction and intrinsic growth rate asymmetries was examined. The growth rate of one cell type was systematically varied, while the other was held constant, and competitive displacement at the biomass periphery was quantified. (iii) In the third scenario, the role of reduced surface friction (representing biosurfactant production) in facilitating the persistence of multiple cell types with different growth rates was examined. Simulations were initialized to have between three and six distinct cell types, each of which was assigned an intrinsic growth rate drawn from a uniform distribution ranging from 0.2 to 1.0 hour^−1^. In total, 297 simulations were performed, comprising 67, 74, 69, and 87 replicates for consortia with three, four, five, and six cell types, respectively. The growth of each consortium was simulated for different surface friction conditions, yielding paired outcomes that allowed direct comparison of quantitative features. During surface-associated growth, cells were color-coded according to their intrinsic growth rate to enable real-time visualization of spatial organization. At the end of each simulation, the richness, Shannon entropy, Simpson diversity, and Pielou’s evenness of cell types were calculated from the spatial abundance distributions for each surface friction. (iv) In the fourth scenario, the hypothesis that reduced surface friction increases the width of the peripheral growth band was tested by dividing the biomass into 720 angular sectors (0.5° resolution) using a sector-based algorithm. For each sector, only cells positioned within a specified distance from the peripheral cell were permitted to grow, thereby isolating the effect of the peripheral growth band width. For all scenarios, simulations were initialized with 2000 cells (1600 in the central region and 400 in the annulus) and were terminated when reaching 30,000 cells to ensure computational tractability. The recorded outputs included cell positions, growth rates, and lineage information, all of which were recorded every 10 timesteps.

### Intermixing index in computational modeling simulations

To quantify spatial intermixing between different cell types in the simulations, an intermixing index (*I*_simulation_) was calculated as follows (*18*, *21*)

$$I_{\text{simulation}}=\frac{1}{N}\sum_{i=0}^{N}\;\frac{\sum_{j=0}^{n\left(i\right)}\;I\left(i,j\right)}{n\left(i\right)}$$

In this equation, *N* is the total number of cells, and *n*(*i*) is the number of neighbors in physical contact with cell *i*. For each pair of cells (*i*, *j*), the indicator function *I*(*i*, *j*) takes the value of 1 if the neighboring cell differs in cell type and −1 if it is of the same cell type

$$I\left(i,j\right)=\left\{\begin{array}{c} 1,\;\text{if cell type}\;\left[i\right]\neq \text{cell type}\;\left[j\right]\\ -1,\;\text{if cell type}\;\left[i\right]=\text{cell type}\;\left[j\right] \end{array}\right.$$

This metric is conceptually equivalent to the spatial assortment parameter and segregation index (*74*). Negative values indicate the dominance of isogenic clusters of cells, whereas positive values indicate enhanced mixing between distinct cell types.

### Statistical analyses

All statistical analyses were performed using IBM SPSS Statistics (version 25). Response variables included quantitative measures of surface-associated populations (as specified in Results), whereas explanatory variables corresponded to experimental conditions or group identities (e.g., presence or absence of exogenous biosurfactants). Differences in response variables between groups were assessed using two-sample two-sided Welch tests, which do not assume equal variances between groups (*75*). For correlation measurements, the Pearson correlation test was used to test for linear relationships, whereas the Spearman rank correlation test was used to test for monotonic but nonlinear associations. When performing statistical tests on the same dataset multiple times, *P* values were adjusted using the Bonferroni correction to control for multiple comparisons (indicated as *P*_BC_). Data distributions were examined using Shapiro-Wilk tests. No significant deviations from normality were observed for any of the datasets. For each analysis, the specific statistical test used, *P* value, and sample size are reported in Results. All graphs and data visualizations were generated using Origin 2025b.
