# Supplementary material for: Biosurfactants direct the evolutionary dynamics of surface-associated microbial systems
Source: Sci Adv. 2026 Sep 25;12(39):eaeh8127. doi: 10.1126/sciadv.aeh8127 (PMC13614376; doi:10.1126/sciadv.aeh8127)
Supplement: Supplementary file 1 [file sciadv.aeh8127_sm.pdf]

Science Advances
AAAS

Supplementary Materials for

Biosurfactants direct the evolutionary dynamics of surface-associated microbial systems

Chujin Ruan *et al.*

Corresponding author: Chujin Ruan, chujin.ruan@xmu.edu.cn; David R. Johnson, david.johnson@eawag.ch

Sci. Adv. **12**, eaeh8127 (2026)
DOI: 10.1126/sciadv.aeh8127

The PDF file includes:

Supplementary Text
Figs. S1 to S11
Table S1
Legend for movie S1

Other Supplementary Material for this manuscript includes the following:

Movie S1

Supplementary Text

In the *CellModeller* biophysical framework, each bacterial cell is represented as a spherocylindrical capsule whose configuration is described by seven generalized coordinates

$$\mathrm{q} = \left(x, y, z, \theta_x, \theta_y, \theta_z, l\right)$$

corresponding to three translational, three rotational, and one axial length degree of freedom. The time evolution of each cell is governed by the linear relationship between the applied impulse F and the resulting change in coordinates Δq, expressed as:

$$\mathrm{M}\Delta\mathrm{q} = \mathrm{F} \tag{1}$$

where M is a generalized drag matrix containing translational and rotational viscous drag coefficients μA and the axial elongation resistance term γ. Conceptually, μA corresponds to the resistance to cellular motion from the viscosity of the external medium. γ is the resistance to cellular growth (e.g. by the intrinsic stiffness of the cell wall), which depends on growth rates. However, since both drag terms are applied linearly, the effective biophysical behavior can be decomposed to rely on a single parameter, the ratio $\mu A/\gamma$, which determines the relative ease of elongation versus displacement.

In the default *CellModeller* implementation (see CLBacterium.py, line 952, function calculate_Ax), equation (1) is internally modified by:

$$\mathrm{M} \rightarrow \gamma^{-1}\mathrm{M} \tag{2}$$

where the elongation resistance γ is factored out, and the coefficient $\mu A \rightarrow \frac{\mu A}{\gamma}$. Additionally, by default μA is fixed to unity (μA = 1.0). This reparameterization simplifies the numerical implementation by reducing the model to a single user-defined parameter γ. However, counterintuitively, when studies vary γ, they effectively alter μA in the inverse. To compare simulations with differing γ, the solution is to rescale the temporal domain, which is possible given that the dynamics are linear. Typically, this results in comparisons of simulations with varying γ not at specific time points, but at cell numbers.

In the present study, we varied μA while keeping γ constant. Since we did not factor out μA, modifications of this drag parameter result in consistent behavior that does not require rescaling of time to directly compare simulations.

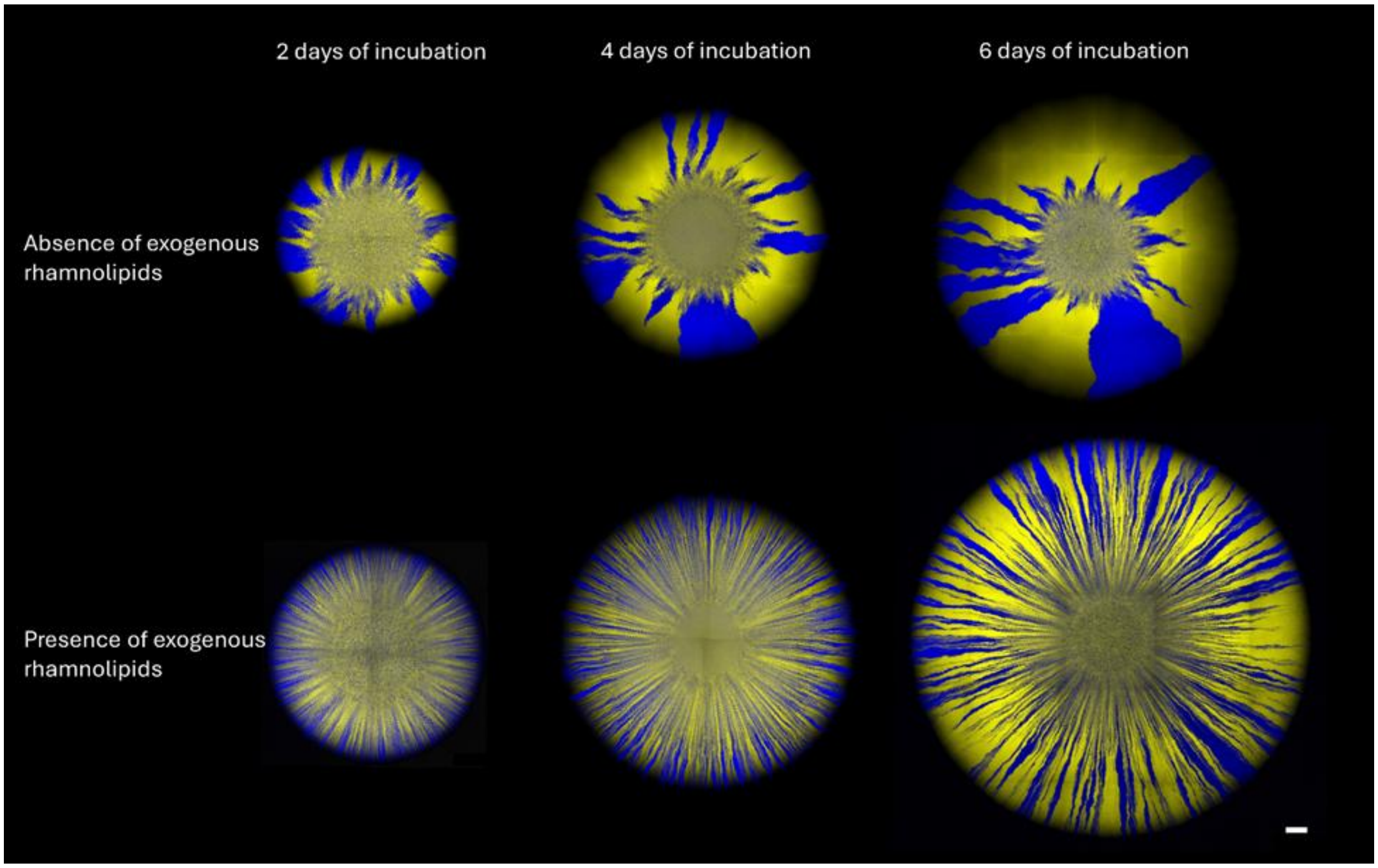

fig. S1. Temporal progression of spatial organization during surface-associated microbial growth. Representative images of the expansion dynamics of *Stutzerimonas stutzeri* consortia grown on agar surfaces in the (top) absence or (bottom) presence of exogenous rhamnolipids after 2, 4, and 6 days of incubation. Fluorescently labelled isogenic populations are shown in false colors (blue and yellow) to aid visual differentiation. Scale bar is 1000 μm.

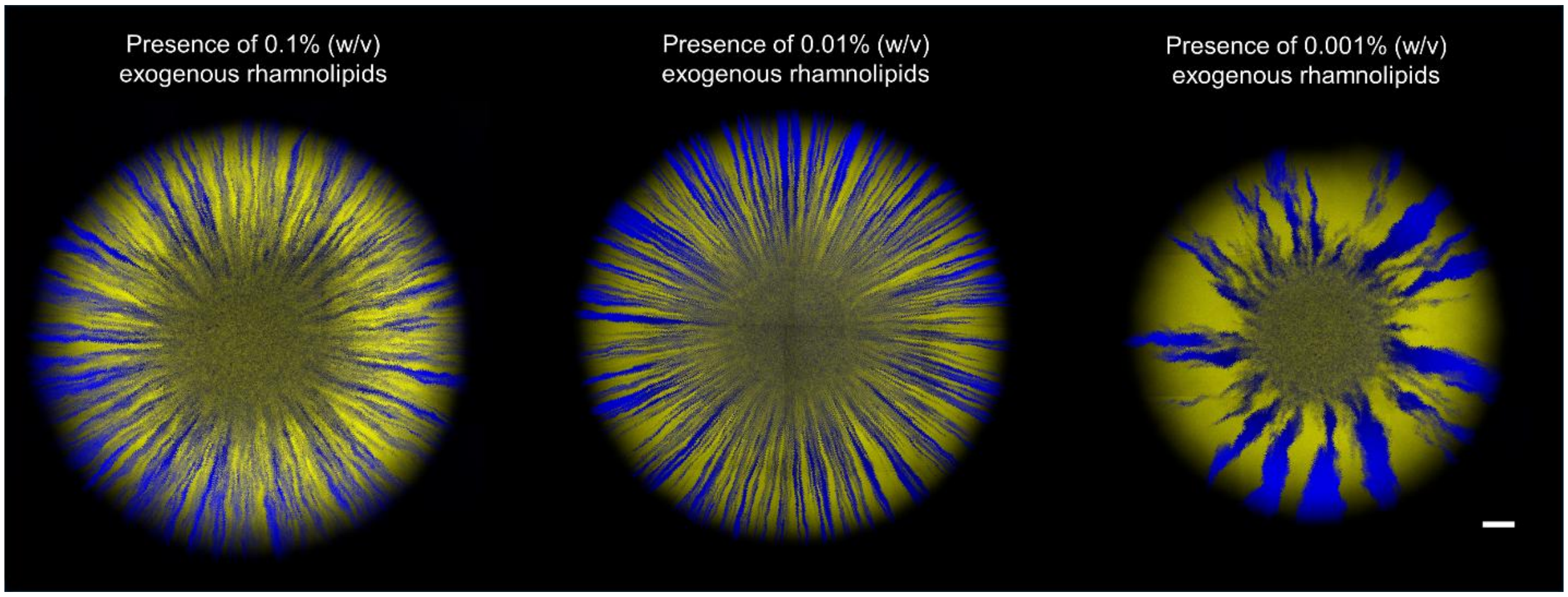

fig. S2. Effect of the concentration of exogenous rhamnolipids on spatial intermixing during surface-associated microbial growth. Representative CLSM images of isogenic fluorescently labeled strains of *S. stutzeri* (strains A1601-egfp and A1601-mcherry) co-inoculated at a 1:1 ratio onto LB agar in the absence or presence of exogenous rhamnolipids at a concentration of 0.001%, 0.01%, or 0.1% w/v. GFP expressing cells are shown in blue, and mCherry expressing cells are shown in yellow. Biomass was imaged after four days of incubation at 30°C. Scale bars are 1000 μm.

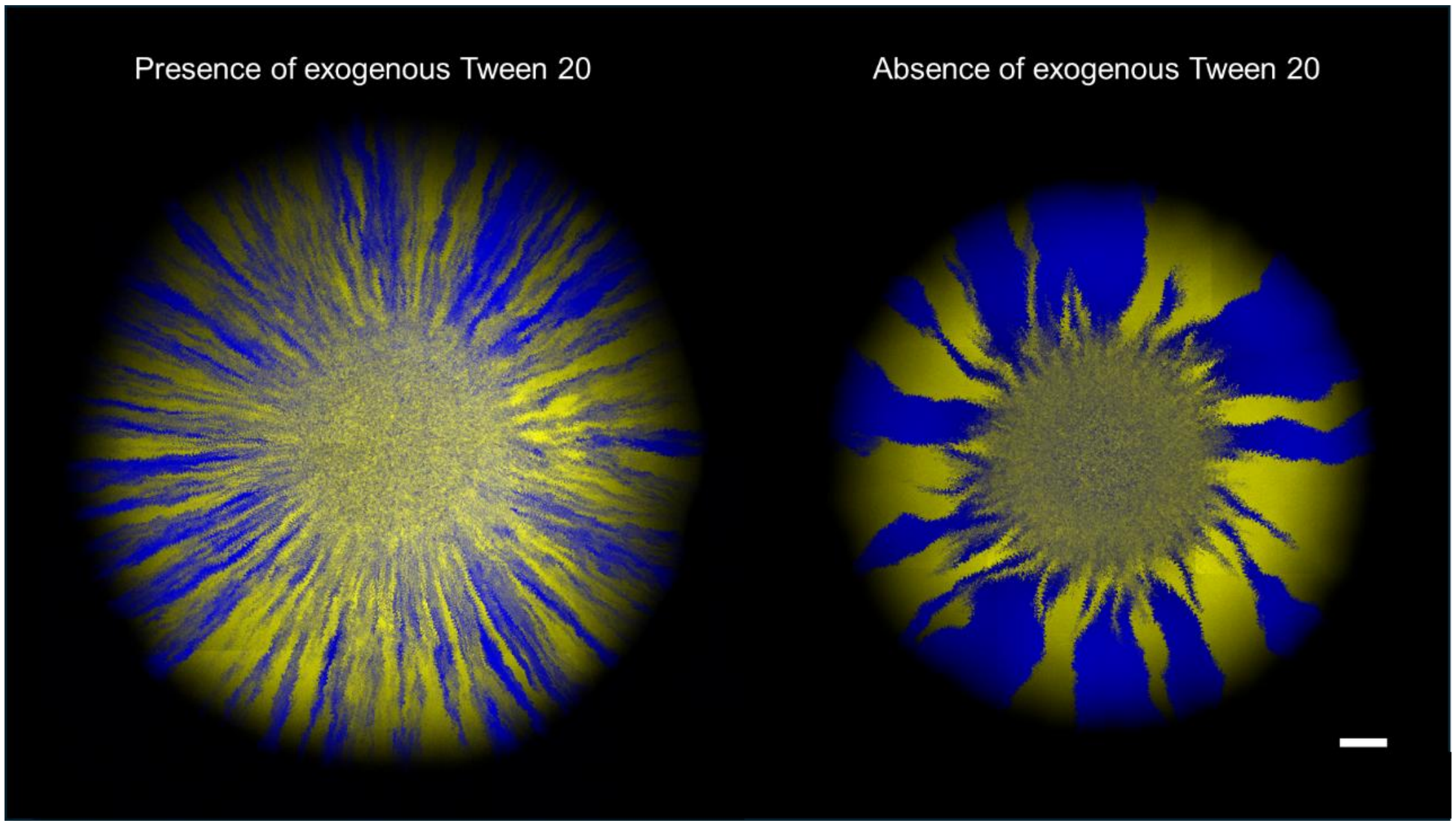

fig. S3. Tween 20 increases the spatial intermixing of different strains during surface-associated microbial growth qualitatively similar to rhamnolipids (see Fig. 1). Representative CLSM images of isogenic fluorescently labeled strains of *S. stutzeri* (strains A1601-egfp and A1601-mcherry) co-inoculated at a 1:1 ratio onto LB agar in the absence or presence of 0.01% w/v exogenous Tween 20. GFP expressing cells are colored blue and mCherry expressing cells are colored yellow. Biomass was imaged after four days of incubation at 30°C. Scale bars are 1000 µm.

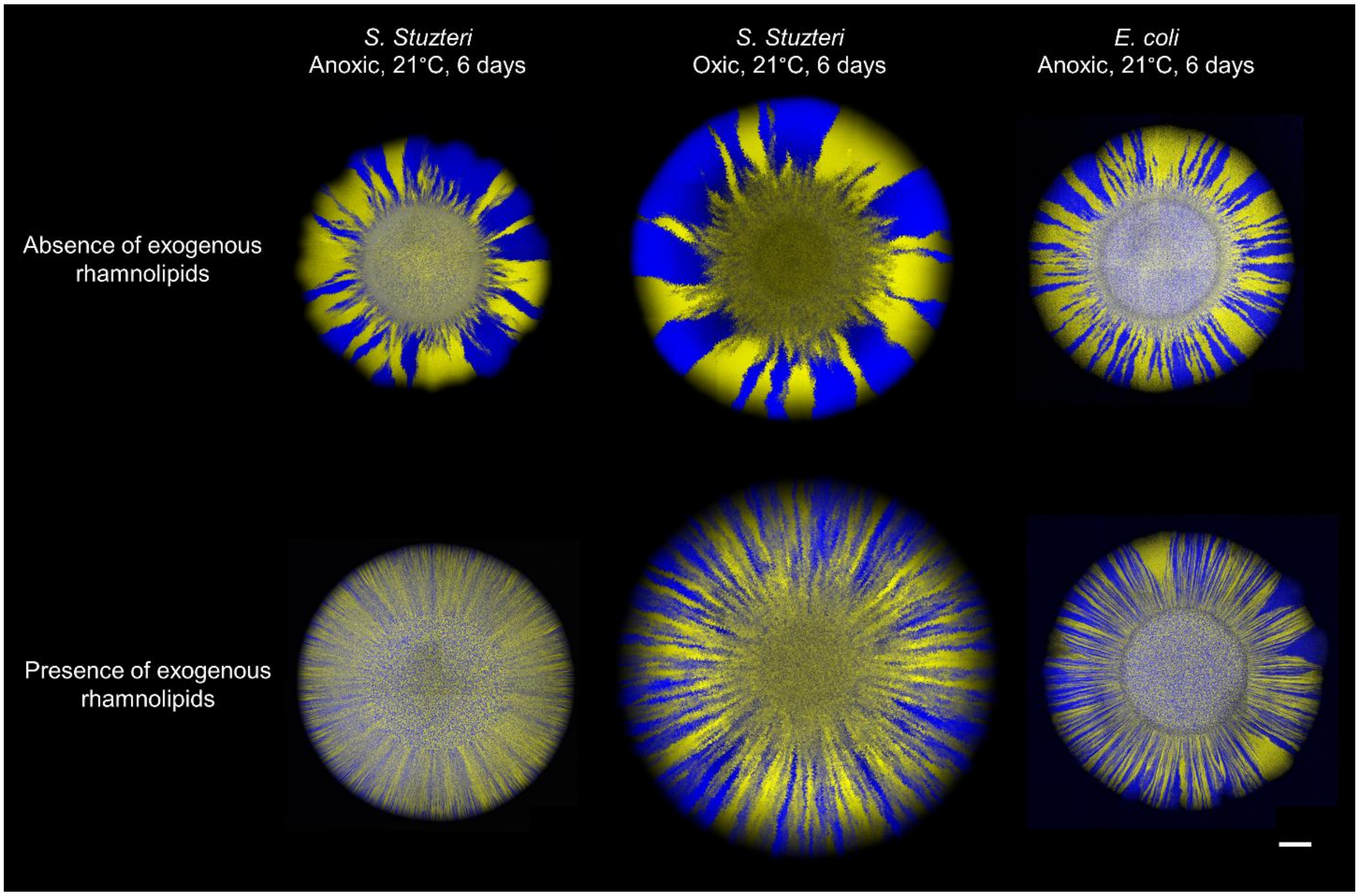

fig. S4. Exogenous rhamnolipids enhance spatial intermixing regardless of oxygen availability and bacterial species during surface-associated microbial growth. Representative patterns of spatial organization of isogenic fluorescently labelled pairs of *S. stutzeri* and *Escherichia coli* strains grown under oxic or anoxic conditions in the absence or presence of exogenous rhamnolipids. False colors denote distinct fluorescently labelled populations (blue and yellow). Scale bar is 1000 μm.

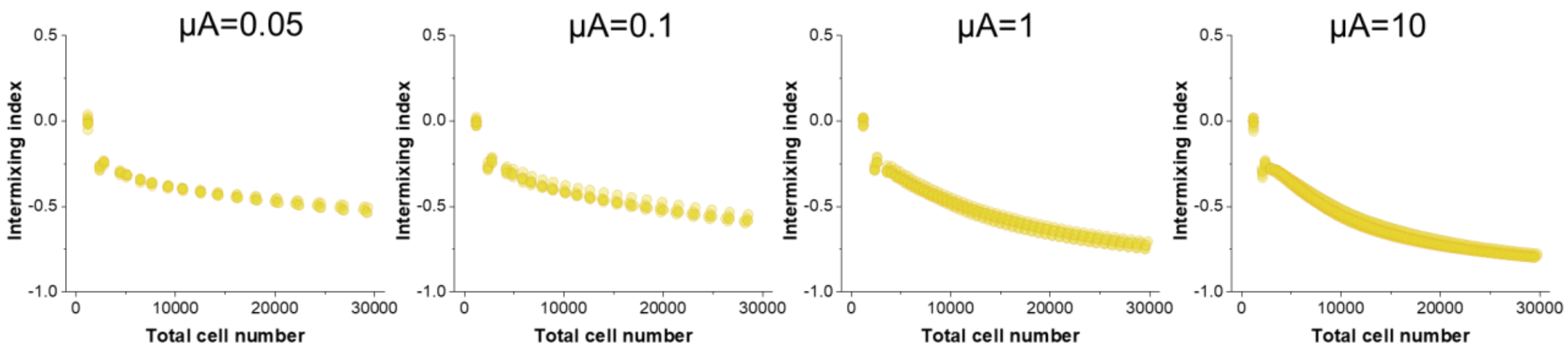

fig. S5. Surface friction modulates spatial intermixing in simulations of surface-associated microbial growth. Individual-based simulation data illustrating the relationship between surface friction (μA = 0.05–10) and the spatial intermixing of two competing lineages during surface-associated community growth. Each panel is for an independent simulation replicate at a given friction level (n = 6 simulations for each friction level).

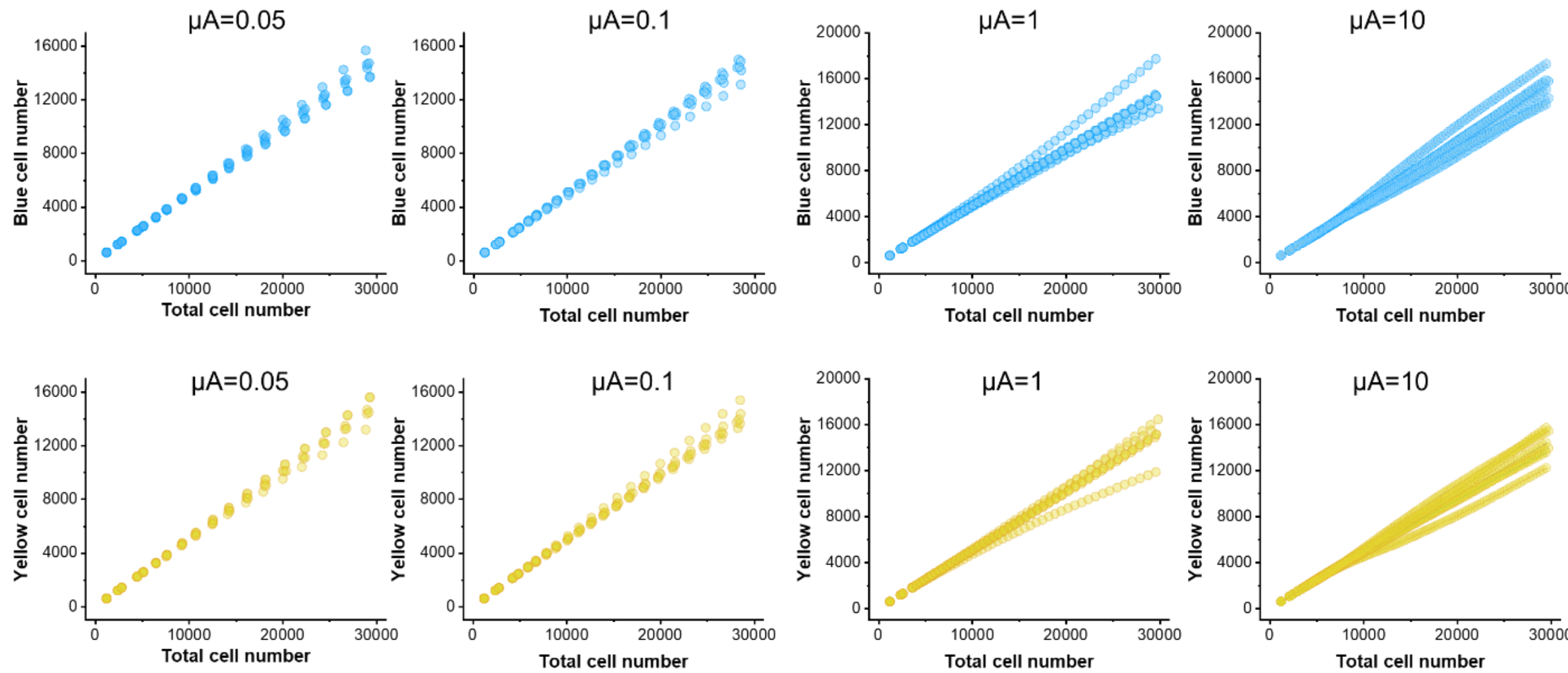

fig. S6. Effect of surface friction on cell numbers in simulations of surface-associated microbial growth. Individual-based simulation data showing the cell number dynamics of two lineages under varying surface friction conditions (μA = 0.05–10). Panels display independent simulation replicates arranged from (left) low to (right) high surface friction. Each panel is for an independent simulation replicate at a given friction level (n = 6 simulations for each friction level).

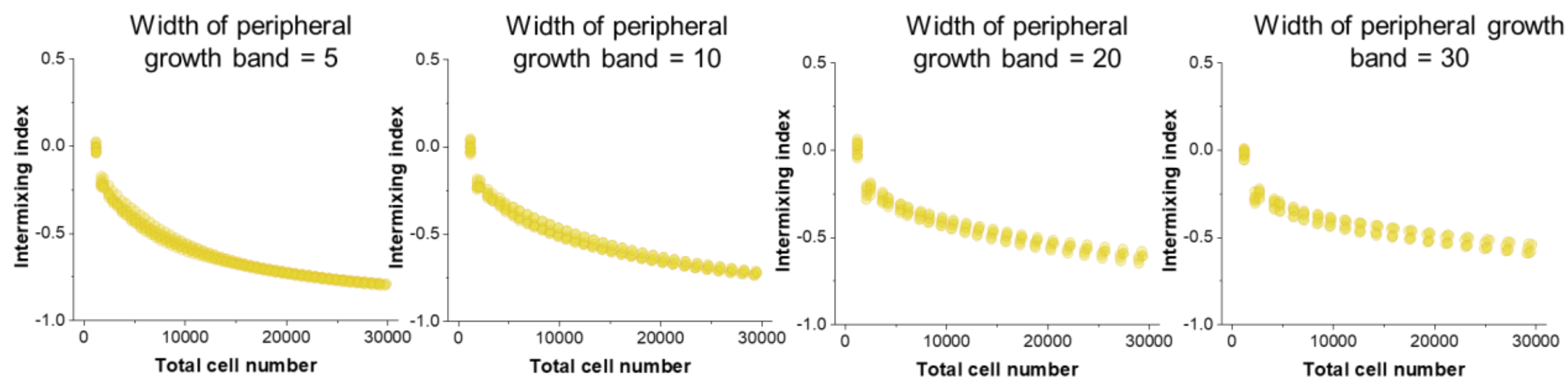

fig. S7. Width of the peripheral growth band determines spatial intermixing in simulations of surface-associated microbial growth. Individual-based simulation data demonstrating how varying the width of the peripheral growth band affects spatial intermixing between two competing lineages. Each panel is an independent simulation replicate for the specified width of the peripheral growth band (n = 6 simulations per width).

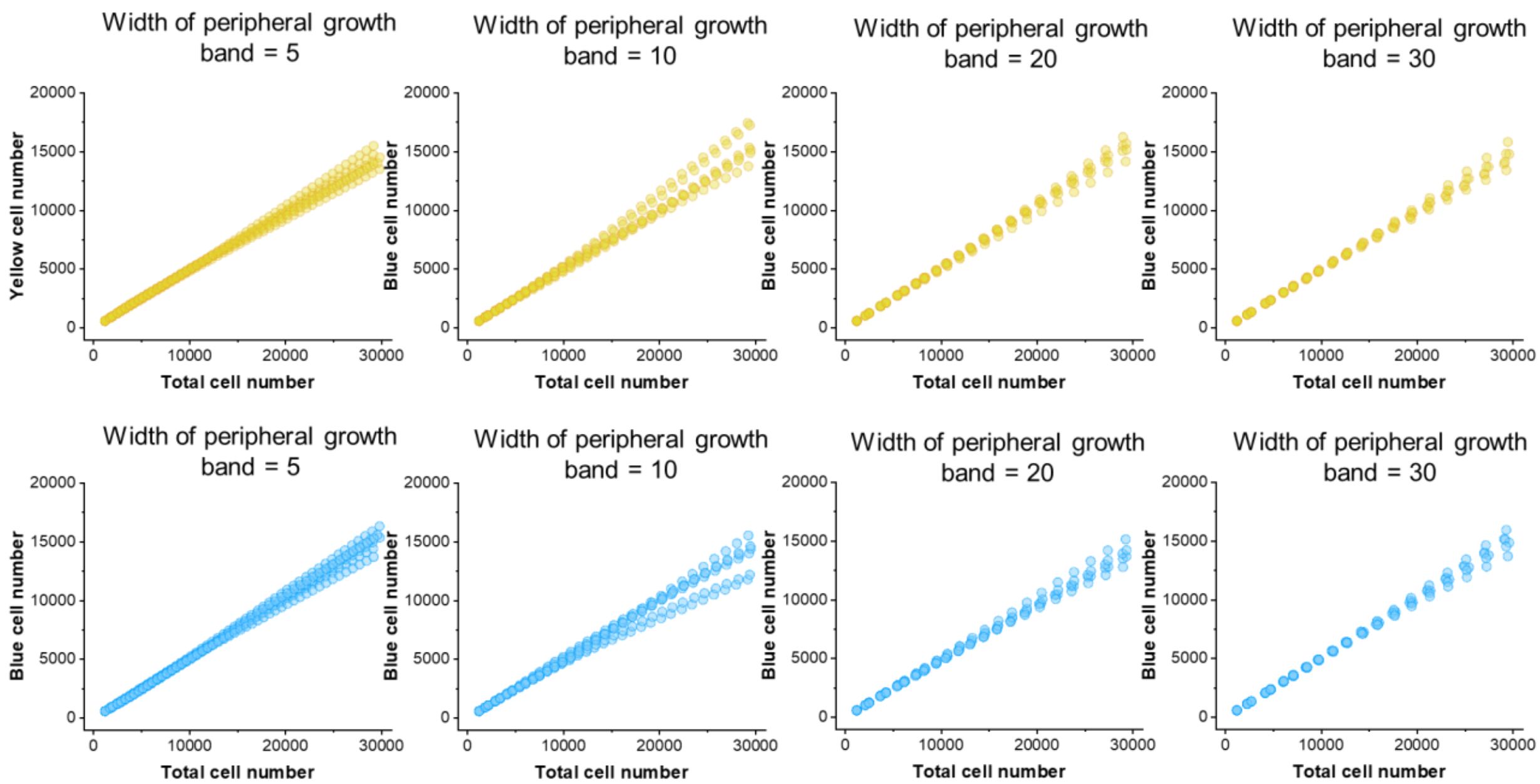

fig. S8 Effect of the width of the peripheral growth band on biomass growth in simulations of surface-associated microbial growth. Individual-based simulation data showing the cell number dynamics of two competing lineages under varying widths of the peripheral growth band. Panels display independent simulation replicates arranged from (left) narrow to (right) wide peripheral growth bands. Each panel is for an independent simulation replicate at a given width of the peripheral growth band (n = 6 simulations for each width).

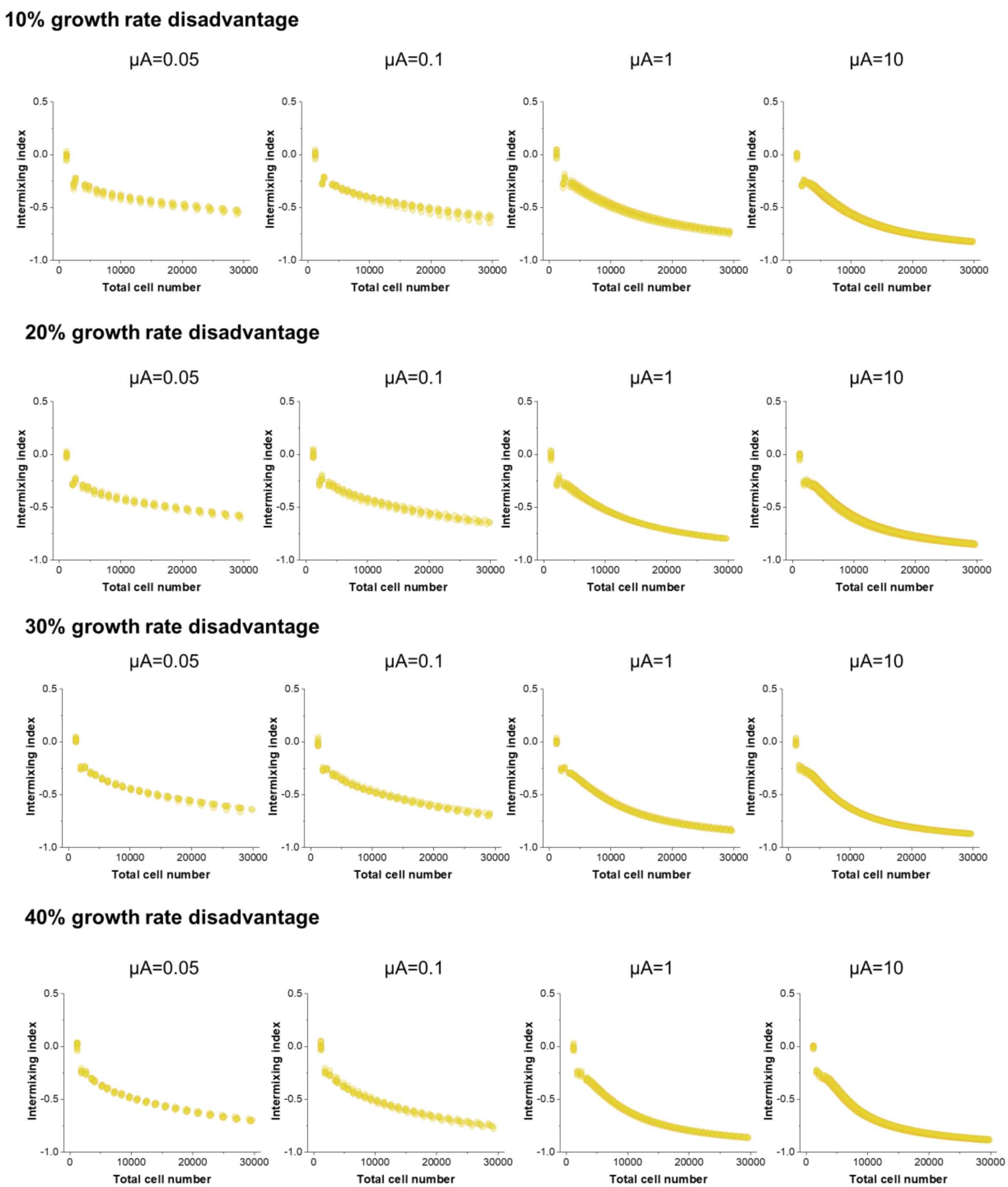

fig. S9. Individual-based simulation data illustrating how growth rate disadvantage and surface friction jointly modulate intermixing during surface-associated microbial growth. Individual-based simulation data showing the intermixing dynamics of subpopulation bearing an intrinsic fitness disadvantage during surface-associated community growth under varying combinations of growth rate disadvantage (10–40%) and surface friction (μA = 0.05–10). Panels display independent simulation replicates (n = 6 simulations for each friction level) arranged by increasing growth rate disadvantage (top to bottom) and increasing surface friction (left to right).

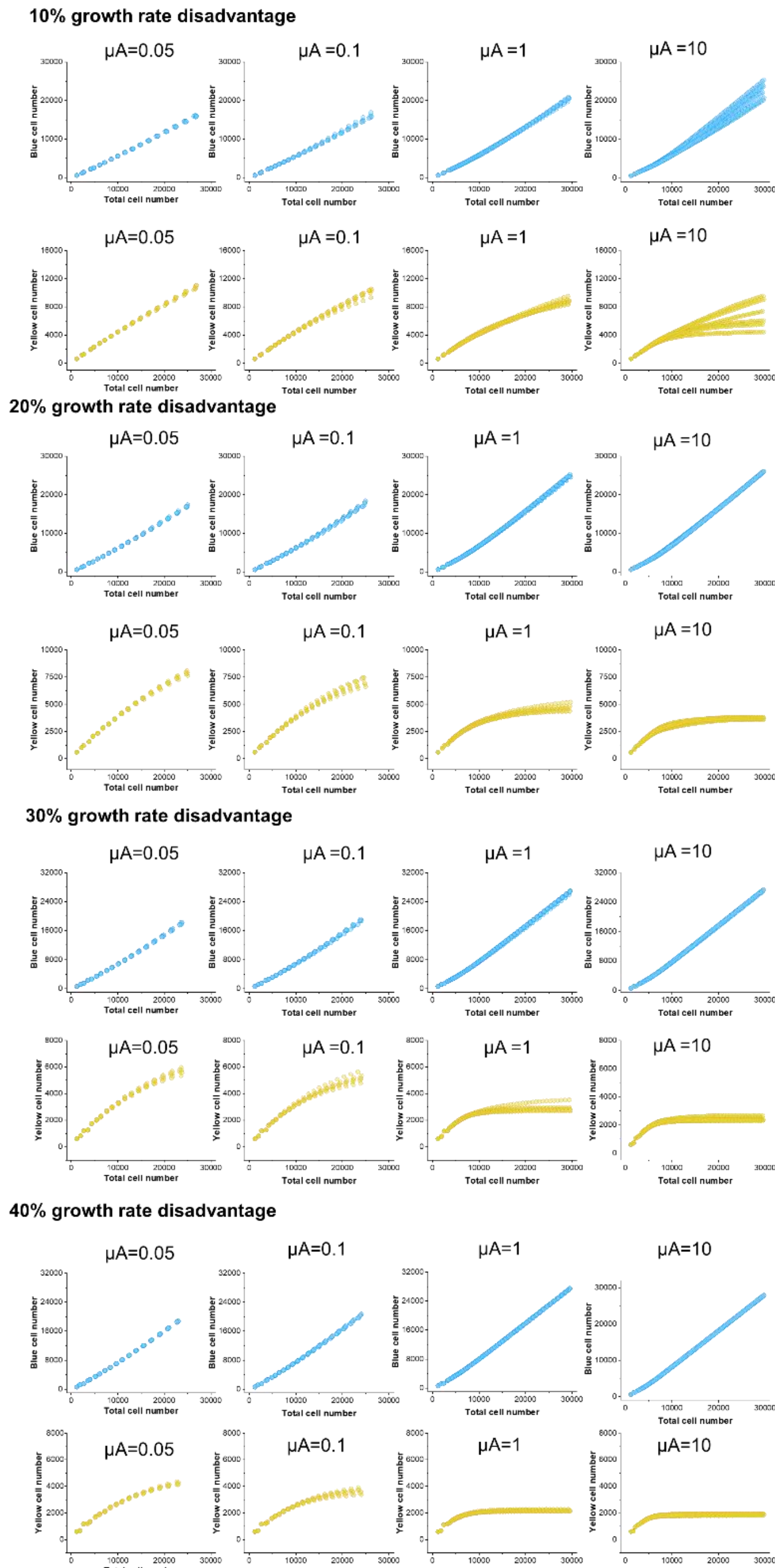

fig. S10. Individual-based simulations showing how the growth rate disadvantage and surface friction affect the abundances of different cell-types. Individual-based simulation data illustrating total cell number (blue) and the biomass of a subpopulation bearing an intrinsic fitness disadvantage (yellow) across combinations of growth rate disadvantage = 10–40% and surface friction (μA = 0.05–10). Panels display independent simulation replicates (n = 6 simulations for each friction level) arranged by increasing growth rate disadvantage (top to bottom) and increasing surface friction (left to right).

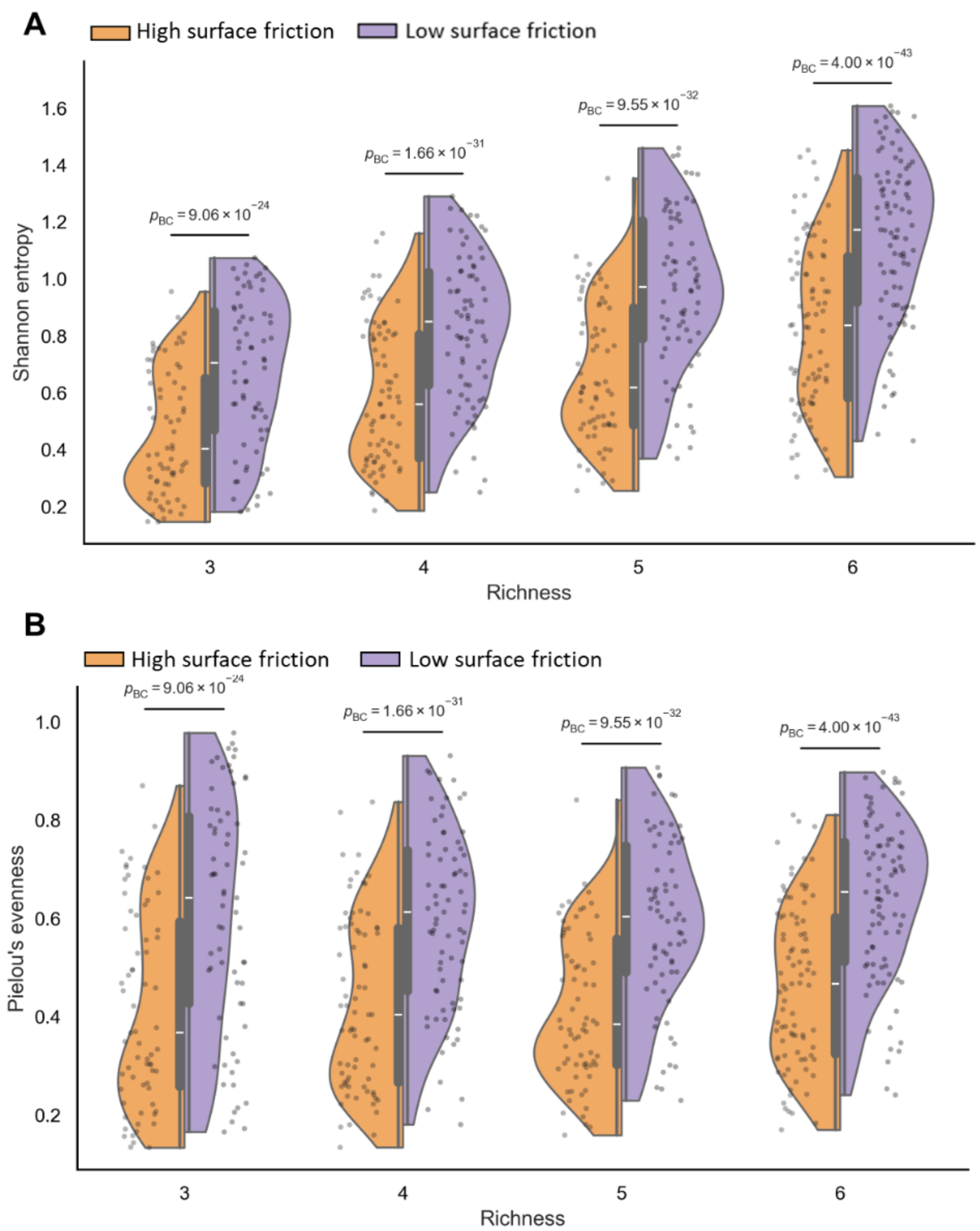

fig. S11. Surface friction determines the maintenance of cell-type diversity. Diversity metrics from individual-based simulations of consortia consisting of three to six distinct cell-types. Each cell-type was assigned a growth rate from 0 to 1 by randomly sampling from a uniform distribution. Violin plots show the distribution of (a) Shannon diversity and (b) Pielou's evenness at different richness levels for high surface friction (yellow; μA = 10) or low surface friction (blue; μA = 0.05). The overlaid boxplots indicate the median and interquartile range, and points are for individual simulations (n ≥ 67). Reported *p* values are from two-sided paired *t* tests with a Bonferroni correction for multiple comparisons.

Table S1. Model parameters used for individual-based simulations.

Parameter	Description	Value / Range
Delta	Mean initial cell length	2
Delta_sig	Standard deviation for the Gaussian-distributed initial target volume	0.4
Rad	Cell radius	0.4
Max_cells	Maximum number of cells allowed in simulation	30,000
μA	Surface friction coefficient in the biophysical module	0.05, 0.1, 1, 10
γ	Axial elongation resistance term	20
R	Radii for the initial seeding region	50
Initial cell number	Initial number of cells	1,200
Peripheral growth width	Width of the peripheral growth band	5, 10, 20, 30

Movie S1. Single-cell time-lapse imaging of *S. stutzeri* growing on agarose pads. Single-cell imaging was performed on 18 mm circular agarose pads composed of 1.5% low-melt agarose and 0.01% (w/v) exogenous rhamnolipids in LB medium. Samples were incubated at room temperature for 24 h prior to imaging. Time-lapse microscopy was carried out on a Nikon inverted microscope using a 100 × oil immersion objective, with bright-field images acquired at 10 min intervals over a 5 h period.
